# Development of a Two Stage Pipeline for Robust Pulmonary Nodule Detection in Consecutive CT Slices

**DOI:** 10.3390/biomedicines14091923

**Published:** 2026-08-27

**Authors:** Jiancheng Wu, Miao Tian, Liaoyuan Zeng, Yujie Xia, Sean McGrath

**Affiliations:** 1 School of Information and Communication Engineering, University of Electronic Science and Technology of China, Chengdu 611731, China; 2 Department of Electronic and Computer Engineering, University of Limerick, V94 T9PX Limerick, Ireland

**Keywords:** computer-aided diagnosis, pulmonary nodule detection, Kalman filter, nodule tracking, maximum intensity projection

## Abstract

**Background/Objectives:** Current pulmonary nodule detection systems often neglect the spatiotemporal continuity inherent in computed tomography (CT) sequences within a single scan. They treat individual slices as independent images and lack mechanisms to recover missed detections. As a result, nodules missed in individual slices cannot be recovered. This study aims to develop a detection–tracking co-design framework that improves both sensitivity and specificity for pulmonary nodule detection. **Methods:** We propose a detection-tracking co-design framework. It couples an enhanced YOLOX detector with a Kalman filter-based tracker in Stage I, to associate nodule candidates across consecutive slices and recover missed detections. Stage II employs cross-slice Maximum Intensity Projection and a lightweight ResNet-50 classifier, to reduce false positives through morphological feature discrimination. **Results:** Systematic ablation studies on the Lung Image Database Consortium and Image Database Resource Initiative (LIDC-IDRI) dataset using the criteria of the Lung Nodule Analysis 2016 (LUNA16) dataset demonstrate the complementary contributions of each component. The complete framework achieves a Competition Performance Metric (CPM) score of 0.9344 and exhibits particular strength in medium-to-high sensitivity regions. **Conclusions:** The proposed detection–tracking co-design provides an interpretable paradigm that balances high sensitivity and improved specificity, offering a promising solution for computer-aided diagnosis of pulmonary nodules.

## 1. Introduction

Lung cancer is a major contributor to cancer-related mortality, as it is among the most frequently diagnosed cancers worldwide. It causes approximately 1.8 million deaths worldwide annually [1,2]. The five-year survival rate differs markedly between patients diagnosed at early versus late stages. Early lung cancer detection can reduce mortality and improve survival outcomes [3,4]. Pulmonary nodules can indicate the presence of early-stage lung cancer. Accurate detection of these nodules is a crucial step in achieving early screening. This enables the provision of curative treatments, such as surgical resection or radiotherapy [4]. Therefore, enhancing both the accuracy and accessibility of early pulmonary nodule detection is necessary. This advancement is crucial for enabling the early detection and clinical management of lung cancer and for ultimately improving patient outcomes.

Low-dose computed tomography (LDCT) has the advantages of reduced radiation dose, fast scanning speed, and sensitivity comparable to standard computed tomography (CT). LDCT is recognized as a promising imaging modality for early lung cancer detection [5]. Its application has enhanced early detection capabilities, allowing interventions to be conducted at a curable stage [1,6,7,8,9]. However, the widespread use of LDCT in average-risk populations has raised concerns regarding overdiagnosis. A recent registry-based study in China reported that the overdiagnosis rate of lung cancer in women reached 50% during 2016–2020, with lung adenocarcinoma overdiagnosis as high as 89% [10]. This highlights the need for CAD systems that not only maintain high sensitivity but also improve specificity to reduce false-positive findings. Examining pulmonary nodules using LDCT generates a vast number of tomographic images. This imposes a heavy burden on radiologists and can lead to visual fatigue. Manual detection is often associated with subjectivity and low reproducibility. These factors contribute to persistently high rates of missed diagnosis.

To address the limitations of manual detection, Computer-Aided Diagnosis (CAD) techniques have been developed. These techniques aim to provide objective and efficient detection references for doctors [11]. Traditional CAD methods primarily relied on handcrafted features to describe pulmonary nodules, such as the Histogram of Oriented Gradients (HOG) [12], Local Binary Patterns (LBP) [13], and the Gray-Level Co-occurrence Matrix (GLCM) [14]. These features were used with simple classifiers for nodule discrimination. Pulmonary nodules exhibit high heterogeneity in their size, shape, density, and location. They can be confused with normal structures such as blood vessels and bronchi in medical images. These inherent characteristics of nodules make it difficult for traditional methods to capture their complex and variable essential traits. Traditional CAD systems often suffer from insufficient detection sensitivity and high false positive rates [15].

Deep learning technology has driven the evolution of CAD systems towards data-driven feature learning. Modern CAD systems based on deep learning can handle diverse medical image analysis tasks, such as classification, detection, and segmentation [15,16,17]. The deep learning architectures have been successfully introduced to deal with the task of pulmonary nodule detection. Variants and specialized networks based on convolutional neural network (CNN) now form the backbone of this field, such as U-Net [18] with its improvements, MV-KBC [19], LNCDS [20], Faster R-CNN [21], and the YOLO series with its variants [22,23,24,25]. Hybrid models have begun to emerge, such as ANN [26] and CNN-LSTM [27]. By automatically learning more discriminative image features, these methods have surpassed the performance bottlenecks of traditional methods in terms of both accuracy and generalizability.

Pulmonary nodule detection methods can be broadly classified into two primary categories, two-dimensional (2D) detection and three-dimensional (3D) detection. Ji et al. [28] proposed a novel YOLOv5-CASP model that incorporates improved Convolutional Block Attention Module (CBAM), enhanced Atrous Spatial Pyramid Pooling (ASPP) module, and an introduced Contextual Transformer (CoT) module to detect the location, size, and shape of pulmonary nodules. Methods that focus solely on single slices fail to capture the contextual connection of adjacent sampling slices. With advancements in computer technology, researchers have discovered that 3D networks can encode richer spatial information. Zamanidoost et al. [29] proposed OMS-CNN, an enhanced 3D Faster R-CNN framework. It employs metaheuristic-optimized multi-scale feature fusion and a combined 3D DCNN for false-positive reduction to improve pulmonary nodule detection. Jian et al. [30] proposed CSSANet for lung nodule detection, which introduces a Group Shuffle Attention (GSA) module to enhance inter-slice feature interaction via channel shuffle and parallel channel-spatial attention within a 3D encoder-decoder framework. However, 3D-CNN-based detection methods suffer from high computational costs and GPU memory consumption, limiting their real-time clinical applications [31]. Moreover, these methods process the CT volume in a single forward pass and do not produce per-slice intermediate states. These methods make slice-wise tracking infeasible in the 3D paradigm [32]. These methods also tend to be “black boxes,” failing to consider the general process of pulmonary nodule diagnosis by clinicians.

To comprehensively consider the information from multiple slices, the detection methods for pulmonary nodule detection can include two stages: (1) candidate nodule detection, and (2) false positive reduction. The first stage aims to detect nodules with high sensitivity, while the second stage further optimizes the detection results by integrating contextual information. Xie et al. [33] proposed an automated pulmonary nodule detection framework based on Faster R-CNN. It adapted the network by introducing dual region proposal networks and a transposed convolutional layer to identify candidate nodule regions. Three models were trained on three distinct slice types, and their outputs were fused to generate results. Finally, a 2D classifier was used to remove false positive nodules. Kanipriya et al. [27] utilized a combination of machine learning and deep learning for pulmonary nodule localization, segmentation, and classification. They employed entropy-based K-means clustering, active contour level set algorithm, and CNN with LSTM to capture temporal information from consecutive CT images, enabling more accurate nodule classification. Zheng et al. [34] combined MIP with CNN for pulmonary nodule detection. This method uses MIP to integrate spatial information into two-dimensional images to distinguish nodules from blood vessels. It processes images of different thicknesses as inputs to the multi-view model and integrates them in the fusion stage. Finally, a classifier was trained to remove false-positive nodules. The performance of pulmonary nodule detection has been improved with the development of the aforementioned deep learning-based methods. Nodules that are missed by the detection network in the first stage cannot be recovered in the subsequent discrimination stage, and are never included in the candidate set for further analysis.

The two-stage method described above also faces significant performance bottlenecks in the false positive reduction stage. The core challenge lies in the fact that the cross-sectional appearances of pulmonary vessels and bronchi in independent 2D slices are visually highly similar to nodules, which can easily lead to algorithmic misjudgments. Regarding the screening of false positive candidate nodules, some studies have focused on processing multi-dimensional features of the input data. Usman et al. [35] employed a strategy utilizing bidirectional maximum intensity projection images, combining 3D CT slices with a self-distillation mechanism to suppress redundant non-nodular structures through multi-scale feature integration. Xiong et al. [36] adopted a model fusion strategy by introducing the Weighted Box Fusion algorithm to integrate the outputs of multiple detection networks, achieving more robust false positive filtering. Another line of research focuses on capturing more complex contextual relationships. Cui et al. [37] proposed an innovative dual-stream network that independently processes raw image features and morphological features across depth scans, and seamlessly integrates them via an attention mechanism to effectively eliminate high-confidence false positive interferences. These false positive reduction approaches generally tend to incorporate fused feature processing of scan sequences during the early candidate nodule detection stage. Further research is required to enhance false positive reduction performance without increasing the burden on the initial detector.

To address the aforementioned issues, this paper proposes a two-stage pipeline for pulmonary nodule detection and false positive reduction. The pipeline is designed to emulate the clinical image reading process. Existing 2D two-stage frameworks lack this capability, as they do not exploit the spatial continuity of nodules across consecutive slices within a single scan. As a result, nodules missed by the detector in individual slices cannot be recovered. Our pipeline fills this gap by tracking the same nodule across consecutive slices within a single CT scan, enabling slice-to-slice association and the recovery of missed detections. In the first stage, an improved YOLOX detector is integrated with a Kalman filter-based tracking strategy. The Kalman filter associates detection boxes belonging to the same nodule across consecutive slices, and recovers nodules missed by the 2D detector in individual slices. In the second stage, cross-slice Maximum Intensity Projection (MIP) and a lightweight classification network are employed to discriminate false positives among the candidate results. By incorporating Kalman filtering and MIP, the pipeline offers interpretability and enables a more transparent analysis of the detection process. The framework achieves a substantial reduction in false alarms while preserving high sensitivity. The primary contributions of this paper are summarized as follows:

1. A detection-tracking co-design framework is proposed for pulmonary nodule detection with missed-detection recovery. The proposed framework couples the improved detector with a Kalman filter-based tracker.
2. Stage I. The improved YOLOX detector, enhanced with GIoU and NWD losses, outputs detection boxes with localization accuracy and inter-slice smoothness. A Kalman filter-based Candidate Nodule Tracking algorithm then associates detections across consecutive slices and recovers missed nodules.
3. Stage II. It employs cross-slice Maximum Intensity Projection and a lightweight ResNet-50 classifier for false positive reduction.

The subsequent sections of this paper are structured as follows: Section 2 introduces the overall architecture of the proposed framework and details the methodology for each stage. Section 3 describes the experimental setup. Section 4 presents and analyzes the experimental results. Section 5 provides a discussion of the findings. Section 6 concludes the paper.

## 2. Methods

### 2.1. The Overall Design of the Proposed Pipeline

This paper proposes a two-stage deep learning pipeline for pulmonary nodule detection and false positive reduction. The framework accepts consecutive CT slices from a single scan as input and outputs a set of localized nodules with associated confidence scores. The core design of the framework emulates the diagnostic logic of clinical radiologists. Radiologists synthesize observations across consecutive slices to form a diagnosis. This work translates the clinical cognitive process into a computable algorithmic pipeline, which enhances CAD systems. This is achieved through the co-design of the detector and the tracker. The pipeline maintains high detection sensitivity while improving the transparency and robustness of the decision-making process.

**Figure 1:**
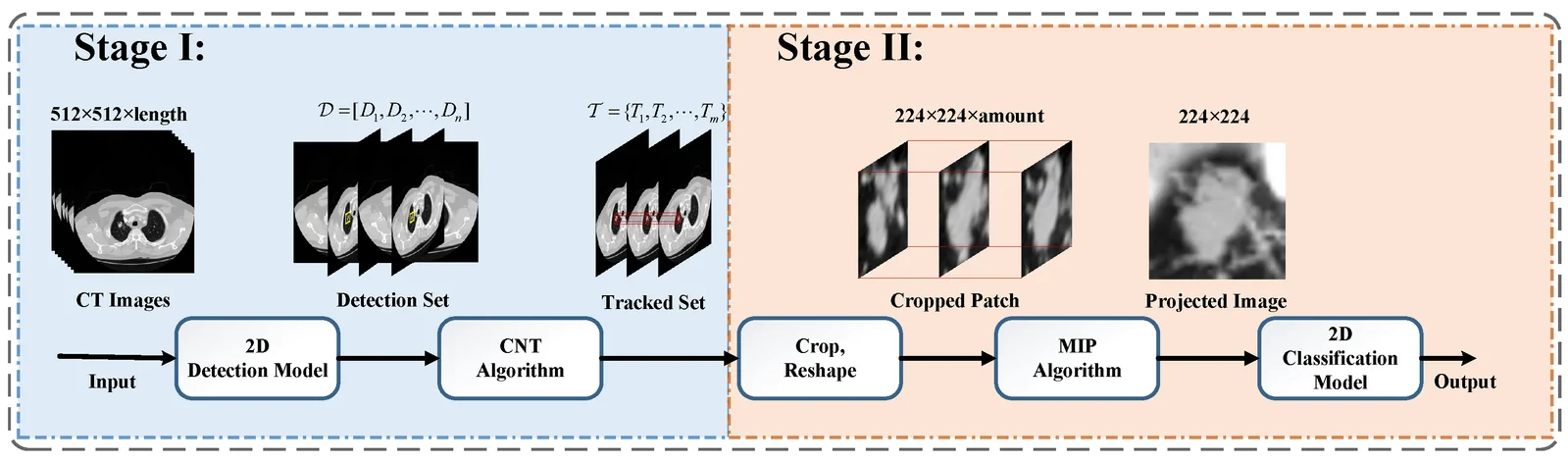
Overall design of the proposed pipeline. The framework accepts consecutive CT slices from a single scan as input and processes them through two stages. Stage I performs 2D detection using the improved YOLOX detector and Kalman filter-based candidate nodule tracking. The tracking function relies on the output of the 2D detector to associate detection boxes belonging to the same nodule across consecutive slices. Stage II applies cross-slice Maximum Intensity Projection to enhance morphological features and employs a lightweight ResNet-50 classifier to reduce false positives.

As shown in Figure 1, the proposed pipeline follows a two-stage architecture. It consists of two coherent and functionally complementary core stages. The first stage performs 2D detection on the CT sequence, and analyzes the spatiotemporal correlations among the detection boxes of consecutive slices. It generates a candidate nodule set with high recall and low redundancy. The second stage then discriminates these candidates by utilizing morphological features, which reduces false positives.

Stage I focuses on generating high-quality candidate nodule set. Unlike conventional approaches that treat each CT slice as an independent image, this stage introduces a critical missed-detection screening mechanism. This mechanism relies on the premise that single pulmonary nodule exhibits spatial continuity across consecutive CT slices. Firstly, each slice is processed by a 2D detection network (as the “2D Detection model” module in Figure 1) for initial detection. Subsequently, a Candidate Nodule Tracking (CNT) algorithm is employed to associate detections across slices. The CNT algorithm explicitly models the spatial continuity of nodules between consecutive slices. It tracks the spatial continuity of each candidate nodule across consecutive CT slices, associating detection targets that belong to the same physical nodule. Based on these associations, the algorithm aggregates all relevant bounding boxes from multiple slices into a unified candidate region. This feature can complete transient missed detections caused by factors such as image noise or low contrast. This design shifts the analytical perspective from processing individual slices in isolation to a coherent examination of the sequential CT series.

Stage II is dedicated to discriminating the candidate regions output by Stage I. It aims to filter out false-positive structures such as vascular cross-sections. This stage employs cross-slice maximum intensity projection (MIP) to compress each candidate region into a feature map. This transformation preserves the morphological characteristics of nodules. These compressed projection images are fed into a lightweight classification network for true-vs-false nodule discrimination.

### 2.2. Stage I: Enhanced 2D Nodule Detector Based on YOLOX

**Figure 2:**
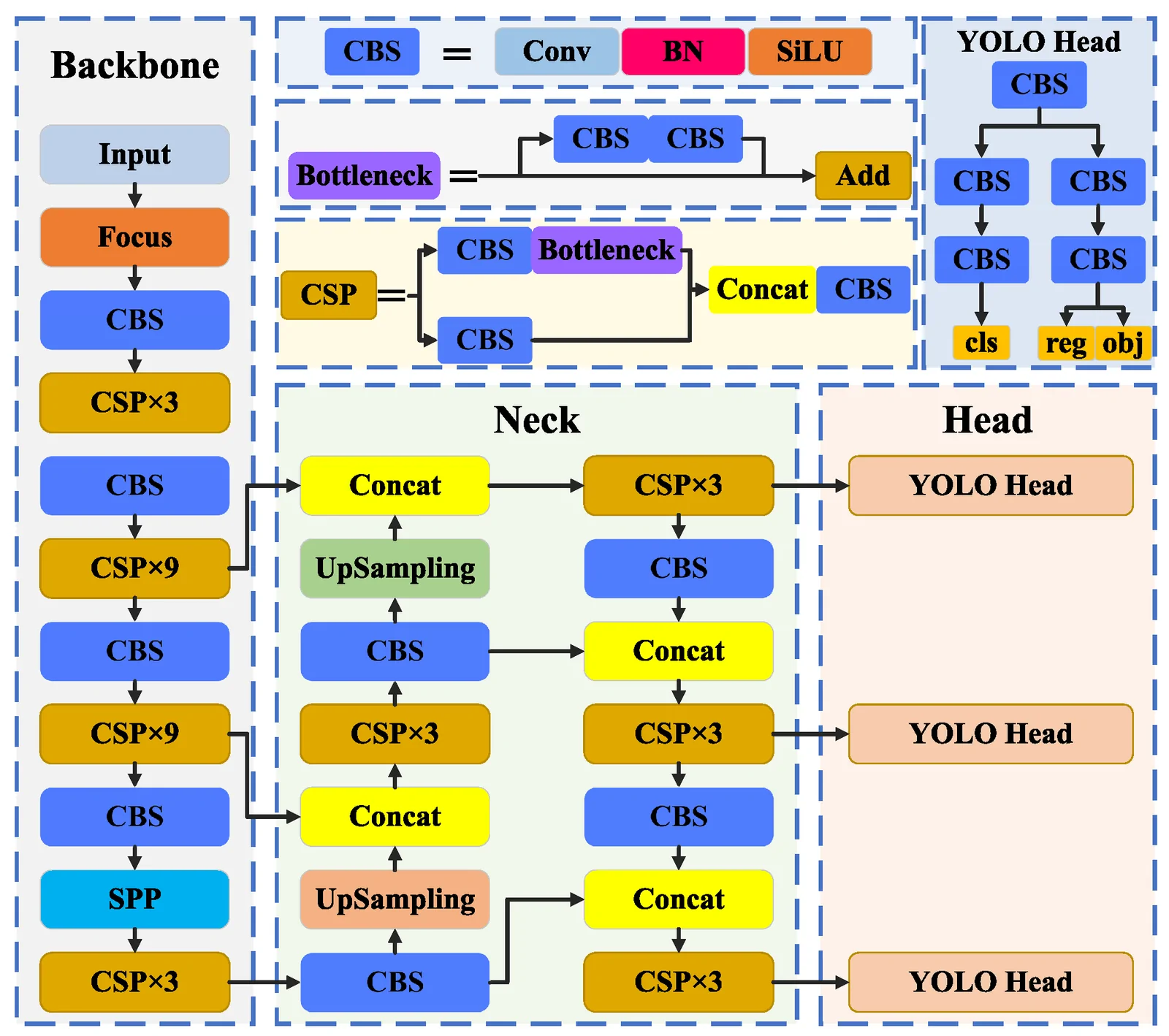
The network architecture of YOLOX. The components are represented by different colors, which are solely used for visual distinction.

We adopt YOLOX [38] as the base two-dimensional detection framework. The overall architecture of YOLOX is illustrated in Figure 2. The model primarily consists of three components: a feature extraction backbone, a feature fusion neck, and a decoupled detection head.

#### 2.2.1. Backbone Network

The backbone utilizes CSPDarknet53. By incorporating the Cross-Stage Partial (CSP) [39] structure based on Darknet [40], it ensures effective gradient flow propagation while increasing network depth. A Spatial Pyramid Pooling (SPP) module is integrated at the end of the backbone to aggregate multi-scale contextual information. The CBS module denotes a sequential stack of Convolution (Conv), Batch Normalization (BN), and SiLU activation. The backbone outputs feature maps at three different resolutions, which contain low-level detailed features and high-level semantic features.

#### 2.2.2. Neck Network

These multi-scale features are fed into the neck network for fusion. The neck is built upon the Path Aggregation Network (PANet) [41], which features top-down and bottom-up bidirectional pathways. This structure facilitates thorough interaction and enhancement between high-level semantic features and low-level geometric features. It constructs a more expressive multi-scale feature pyramid.

#### 2.2.3. Detection Head

YOLOX adopts a decoupled head design. It allocates the classification (cls) task and the bounding box regression (reg) task to two separate lightweight sub-networks for parallel processing. The regression branch also outputs an objectness (obj) score, which indicates the confidence of an object’s presence within the predicted box. This approach mitigates potential conflicts between the two tasks commonly found in traditional coupled heads.

YOLOX employs an anchor-free direct prediction approach and incorporates the Simplified Optimal Transport Assignment (SimOTA) dynamic label assignment strategy. This strategy dynamically assigns the most suitable positive samples to each target during training. The assignment is based on the matching cost between predicted and ground-truth boxes. The original YOLOX-X model has 99.1 million parameters and 281.9 GFLOPs at 640×640 input [38]. Since our experiments use 512×512 input, we report the parameter count and FLOPs measured via the thop profiling library at this resolution: 98.996 million parameters and 180.17 GFLOPs. The minor difference in parameter count arises from the WFF-FPN module replacing the original PANet without introducing extra parameters. The GIoU and NWD losses only modify the training objective and do not affect inference-time computation. Owing to its anchor-free design and the Simplified Optimal Transport Assignment (SimOTA) dynamic label assignment strategy, it is particularly well-suited for detecting small targets like pulmonary nodules that exhibit variable sizes and morphologies. It was selected as the foundational detection model for this research.

The original neck network of YOLOX employs PANet as its feature fusion module. It utilizes a dual-path design comprising both bottom-up and top-down pathways. The PANet uses 1×1 convolutions (as the “Concat” module) to adjust the channel dimensions during feature fusion. This process is followed by the direct concatenation or element-wise addition of the adjusted features. This fusion method treats all input features equally. It fails to consider that features from different scales should contribute differently to the fusion result. This oversight can lead to redundancy of feature information or an imbalance in the fused representation.

**Figure 3:**
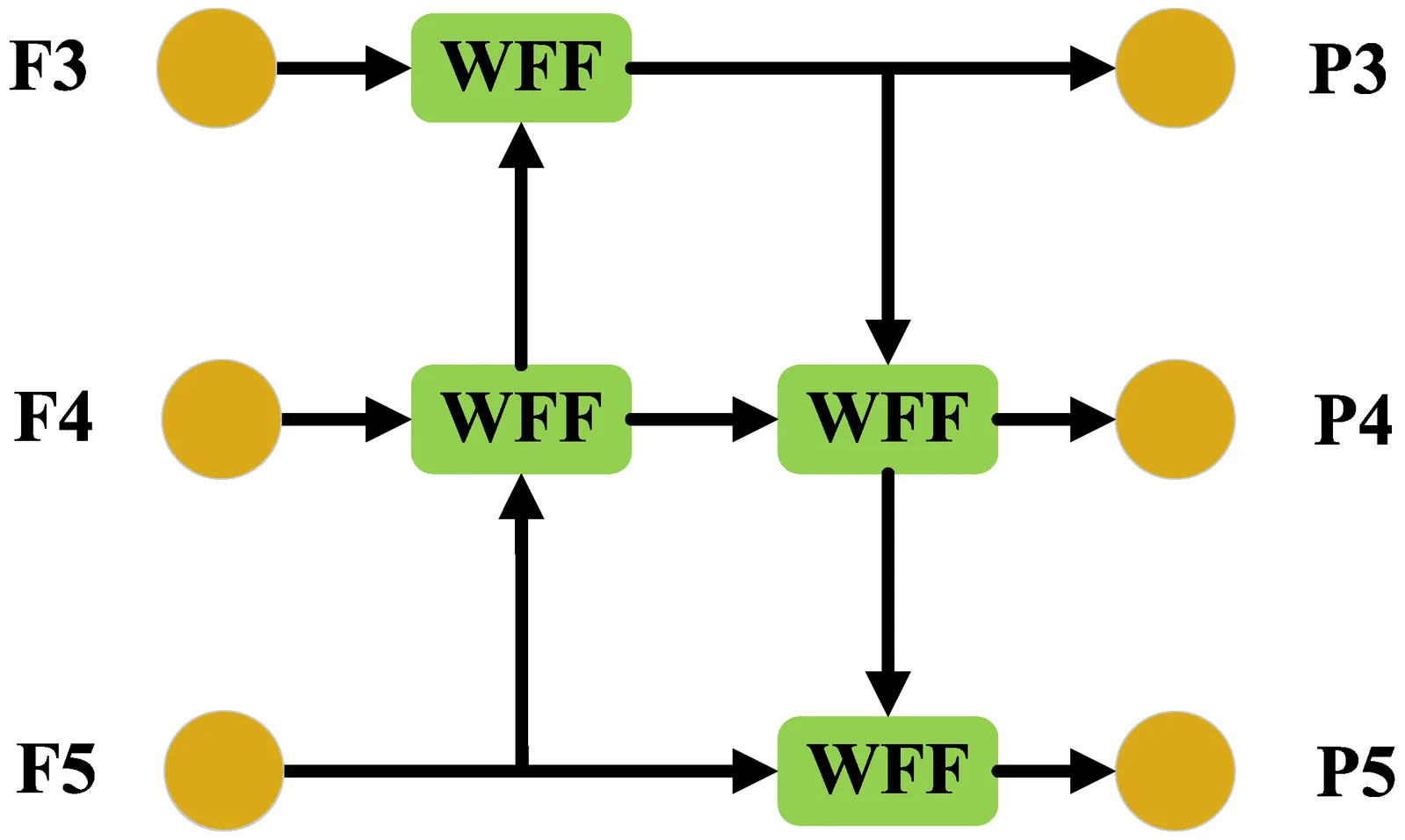
Simplified structures of WFF-FPN. The F3, F4, F5 are the three feature maps output by the main network. The arrows indicate the data flow direction, simplifying the components that pass through. P3, P4, P5 are the outputs of the WFF-FPN.

The Bidirectional Feature Pyramid Network (BiFPN) [42] improves upon PANet. It introduces learnable weights to represent the importance of different input features. BiFPN incorporates additional skip connections on top of the PANet architecture. Our work draws inspiration from the weighted fusion concept of BiFPN but simplifies its structure. We construct a weighted feature fusion (WFF) module (as shown in the green box of Figure 3). We then propose a Weighted Feature Fusion-Feature Pyramid Network (WFF-FPN) to enhance the neck network of YOLOX. WFF-FPN is built upon the PANet architecture by replacing its original concat modules with the proposed WFF module. WFF-FPN omits the intermediate skip connections present in BiFPN. It retains the core mechanism of weighted fusion for adjacent layers. This ensures effective multi-scale information flow while reducing computational complexity.

The improved neck network, WFF-FPN, utilizes learnable weights to modulate the weighted combination of feature maps. This modulation enables the network to adaptively learn the importance of features from different branches. WFF-FPN can dynamically emphasize more critical features during fusion. The calculation of feature map weights in WFF-FPN is shown in (1).

(1) $$\omega_{i}=\frac{\omega_{i}}{\sum_{j}\left(\omega_{j}+\epsilon \right)}$$

where $\omega_{i}$ represents the importance weight of the *i-th* branch feature, with values ranging from 0 to 1. The weights are initialized to 1, assigning equal importance to all input branches at the start of training. ϵ=0.0001 is used to ensure numerical stability during the weight learning process.

The overall loss function of YOLOX comprises three components, the bounding box regression loss $L_{reg}$, the localization loss $L_{obj}$, and the classification loss $L_{cls}$. The overall loss function of YOLOX is shown in (2).

(2) $$L=L_{reg}+L_{obj}+L_{cls}$$

The loss $L_{obj}$ and $L_{cls}$ follow the standard design of YOLOX, as shown in (3) and (4).

(3) $$L_{obj}=-\beta \cdot \sum_{i=0}^{N\times N}\log\left(P_{i}^{\prime }\right)-\left(1-\beta \right)\cdot \sum_{i=0}^{N\times N}\log\left(1-P_{i}^{\prime }\right)$$

(4) $$L_{cls}=-\gamma \cdot \log\left(P_{i}^{\prime \prime }\right)-\left(1-\gamma \right)\cdot \log\left(1-P_{i}^{\prime \prime }\right)$$

where the hyperparameter β∈[0,1] is used to balance positive and negative samples in the binary cross-entropy loss. $P_{i}^{\prime }$ represents the probability that the *i-th* feature within the predicted box is a pulmonary nodule. Here, γ∈[0,1], $P_{i}^{\prime \prime }$ represents the probability of the *i-th* predicted box belonging to the class of pulmonary nodule.

The original YOLOX regression loss is based on the standard Intersection over Union (IoU) loss. When handling pulmonary nodules that typically appear as small, low-contrast lesions, this loss exhibits insufficient gradient sensitivity to center point offsets. This often leads to inaccurate localization or missed detections. To address these issues, we introduce a combined regression loss. It is formulated as a weighted sum of the Generalized IoU (GIoU) loss and the Normalized Wasserstein Distance (NWD) loss, as defined in (5).

(5) $$L_{reg}=\alpha \cdot L_{GIoU}+\left(1-\alpha \right)\cdot L_{NWD}$$

where α=0.8 controls the weighting between the GIoU loss and the NWD loss in the combined regression loss.

The $L_{GIoU}$ is defined as shown in (6):

(6) $$L_{GIoU}=1-\frac{box_{A}\cap box_{B}}{box_{A}\cup box_{B}}-\frac{\left|box_{C}-\left(box_{A}\cup box_{B}\right)\right|}{convex_{C}}$$

where $box_{A}$ and $box_{B}$ represent the predicted bounding box and the ground truth box. $convex_{C}$ denotes the smallest convex hull that encloses both $box_{A}$ and $box_{B}$.

The $L_{NWD}$ loss is designed to enhance the model’s localization sensitivity to small targets, as shown in (7). It models bounding boxes as two-dimensional Gaussian distributions. It measures the similarity between the bounding boxes through the computation of the Normalized Wasserstein Distance (NWD) [43].

(7) $$L_{NWD}=1-NWD\left(box_{A},box_{B}\right)$$

### 2.3. Stage I: Candidate Nodule Tracking and Fusion Based on Kalman Filtering

**Figure 4:**
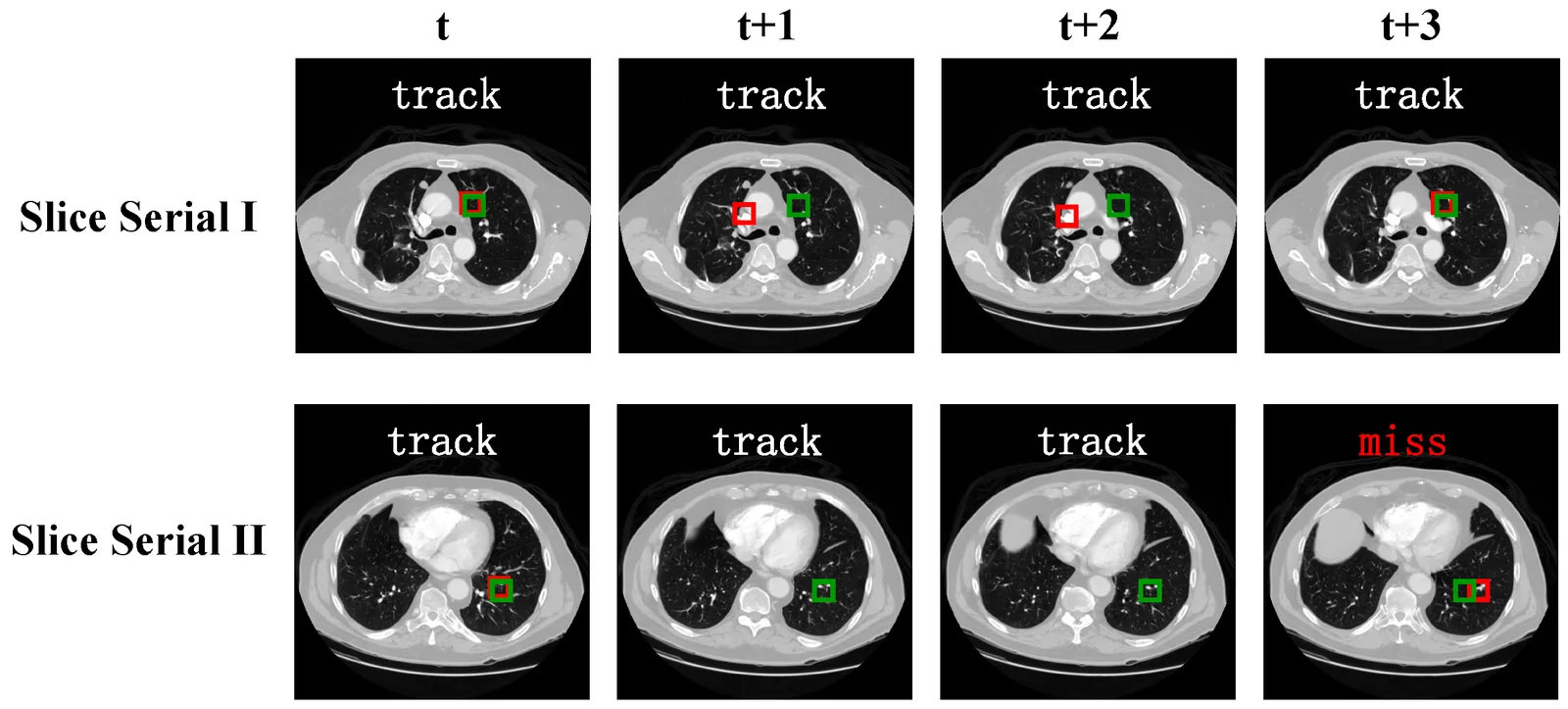
Comparison of centroid distance-based tracking for detected and missed objects. The red boxes denote the raw 2D detection outputs. The green boxes represent the tracked boxes obtained via centroid-distance association. Row I and Row II each show a sequence of four consecutive CT slices. Row I shows a case where the nodule is detected in the first and last slices. The intermediate missed detections (at t+1 and t+2) can be recovered by centroid-distance association. Row II shows a case where the nodule is missed at the sequence boundary (at t+3), making re-identification impossible via distance-based association alone.

The two-dimensional detection model is susceptible to interference from complex backgrounds such as blood vessels and bronchi. Its output bounding boxes may contain genuine nodules or may erroneously select these interfering structures. If a true nodule is detected at both the beginning and the end of a continuous CT sequence but missed in intermediate slices, the centroid distance between bounding boxes can serve as an association metric. This enables cross-slice association and aggregation of detections belonging to the same nodule. However, if a nodule is missed at a critical slice, such as the sequence boundary, this distance-based approach also lacks sufficient reference for association, making re-identification impossible, as illustrated in Figure 4.

To address the limitations of independent slice detection, we incorporate the idea of dynamic object tracking, and propose the CNT algorithm. The core of the CNT algorithm is to treat the CT sequence via the scanning direction (Z-axis) as a temporal series. Each candidate nodule is modeled as a dynamic target moving on the two-dimensional slice plane. The CNT algorithm can associate the same nodule in different CT slices. It employs the established trajectory model to predict the nodule’s state in slices where detection fails. Additionally, the trajectory model is used to complete the state estimation for these missed detections.

**Figure 5:**
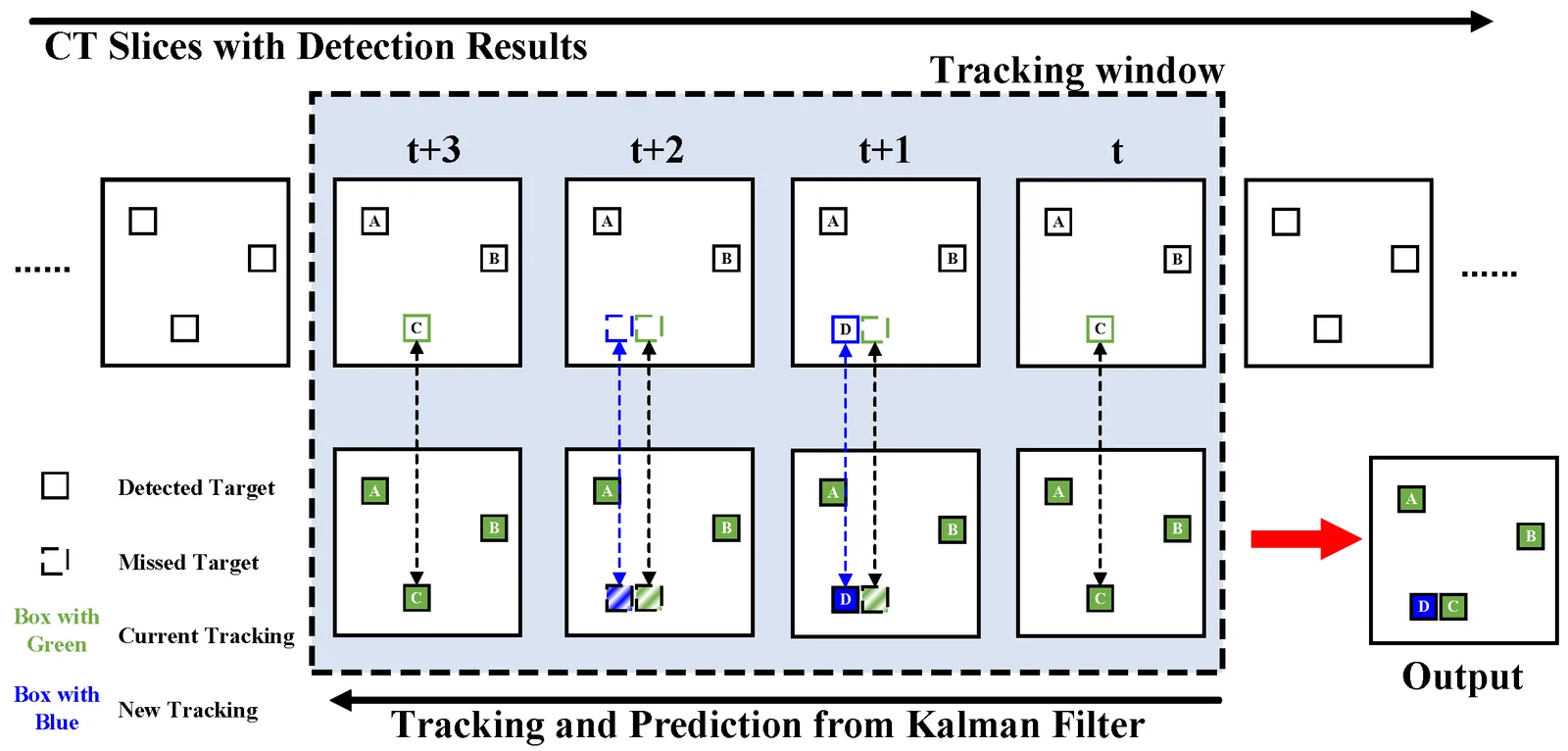
Schematic diagram of operation for CNT algorithm on Kalman filtering. The light blue rectangle in the middle represents the tracking window. The top row presents an ordered CT image sequence (white squares). The small frame inside each square denotes the detection result of the 2D detection network for that slice. A solid frame indicates that the detector has a candidate in that slice. A dashed frame indicates a missed detection. The blue solid box at t+1 in the top row marks a newly detected candidate. The green boxes represent other candidates currently being tracked, with Kalman filter predictions ongoing. The bottom row illustrates the tracking recovery process. Boxes filled with color represent the tracked boxes recovered by the CNT algorithm. The diagonally hatched boxes with alternating colored and white stripes indicate the recovered candidate boxes. Detection boxes belonging to the same candidate across different slices are aggregated into a single representative result. The Output section on the right presents the final tracked results for four targets (A, B, C, and D). The ellipsis indicates omitted preceding and following slices. Only a representative segment of the CT series is shown for clarity.

Figure 5 illustrates the operational workflow of the CNT algorithm. When the detector identifies a new candidate object in the current slice (e.g., the blue solid frame *D* in the t+1 slice), the CNT algorithm initializes a new tracker for it. Each tracker contains an embedded Kalman filter, which is responsible for estimating the state of that candidate nodule across subsequent consecutive CT slices. If a detection miss occurs in later slices (as indicated by the dashed frames at t+1 and t+2), the CNT algorithm invokes the Kalman filter. The filter predicts the bounding box position in the current slice based on the target’s prior motion state. When the prediction meets specific confidence criteria, this predicted box is used to fill the detection gap in the tracking window caused by the miss.

The CNT algorithm employs the Hungarian algorithm for data association between slices. The association cost between a detection and an existing tracker is defined as the Euclidean distance between their bounding box centers. A match is accepted only when this distance falls below a threshold of 5 pixels. This process effectively associates and aggregates redundant detection boxes that belong to the same physical nodule but are scattered across adjacent slices. The algorithm outputs one most representative bounding box for each tracked nodule. As shown in the “Output” section at the bottom of Figure 5, after processing by the CNT algorithm, the sequence outputs four aggregated nodule detection results, corresponding to targets *A*, *B*, *C*, and *D*.

**Figure 6:**
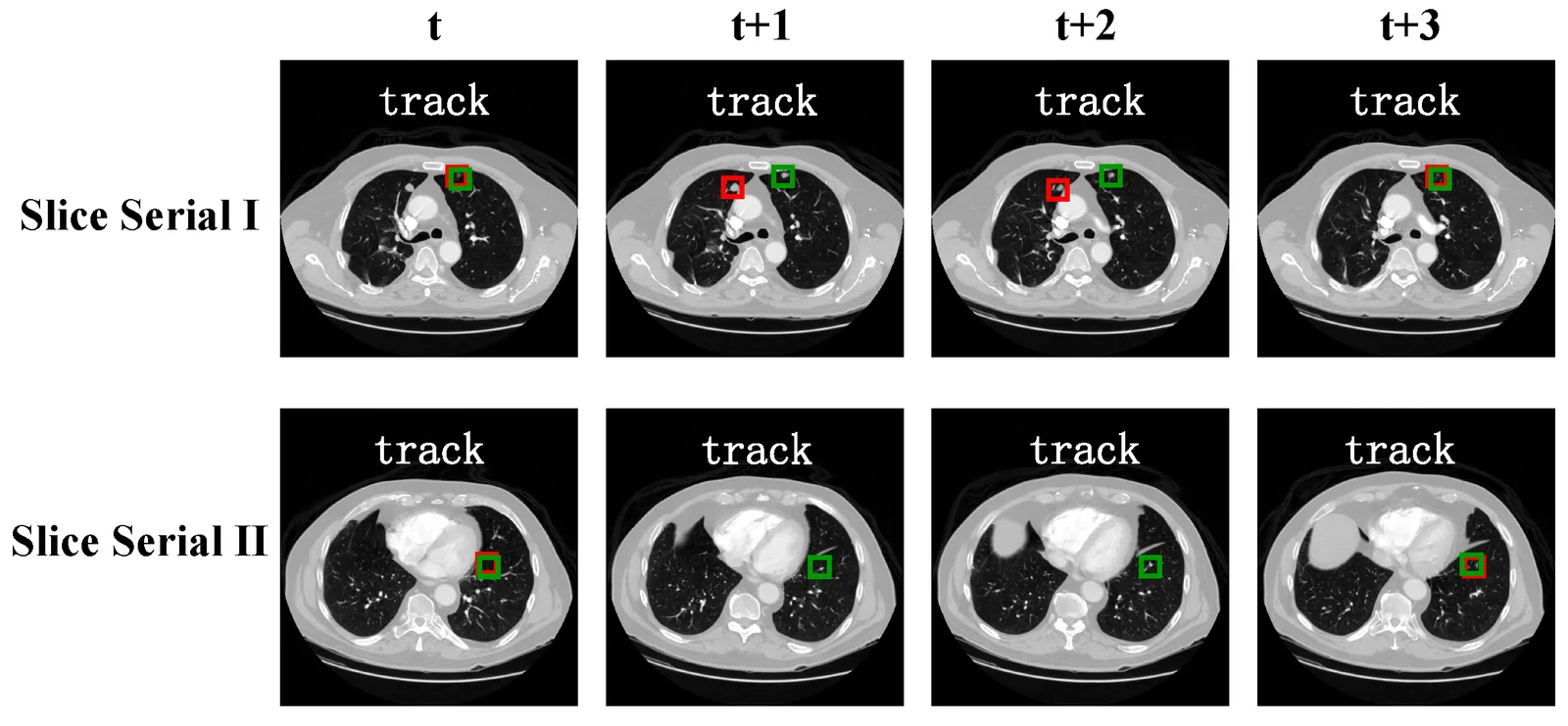
Comparison of CNT algorithm tracking for detected and missed objects. The red boxes denote the raw 2D detection outputs. The green boxes represent the tracked boxes obtained via the CNT algorithm. Row I and Row II each show a sequence of four consecutive CT slices.

As shown in Figure 6, this robust tracking method based on Kalman filtering effectively addresses the shortcomings of the simple IoU-based association. It maintains reliable tracking continuity even when detections are missed at arbitrary slice positions.

To formalize the prediction and update steps of the Kalman filter within each tracker, we define the mathematical representation of a nodule’s state and its observation. The state of a candidate nodule on the *k*-th slice is defined as a five-dimensional vector $z_{k}$, called observation vector, as shown in (8).

(8) $$z_{k}={\left[c_{x},c_{y},w,h,confidence\right]}^{T}$$

where $\left(c_{x},c_{y}\right)$ represents the center coordinates of the bounding box, *w* and *h* denote its width and height. Confidence is the confidence coefficient output by the detection model.

The Stage I detection network (the improved YOLOX model) outputs the raw coordinates of a bounding box as $D_{k}$, as shown in (9). The output $D_{k}$ is transformed into the observation vector $z_{k}$ through the (10) and (11):

(9) $$D_{k}=\left(X_{min},X_{max},Y_{min},Y_{max}\right)$$

(10) $$c_{x}=\frac{X_{min}+X_{max}}{2},c_{y}=\frac{Y_{min}+Y_{max}}{2}$$

(11) $$w=X_{max}-X_{min},h=Y_{max}-Y_{min}$$

where $X_{min}$, $X_{max}$, $Y_{min}$, and $Y_{max}$ denote the left, right, top, and bottom pixel coordinates of the predicted bounding box, respectively.

The Kalman filter is based on a linear Gaussian model. It has been applied in medical image sequence tracking, such as volumetric imaging during radiation therapy [44]. It recursively estimates the state through two steps: prediction and update. The state prediction equation is given by (12) and (13).

(12) $${\hat{x}}_{k}^{-}=A{\hat{x}}_{k-1}^{-}+Bu_{k-1}$$

where ${\hat{x}}_{k}^{-}$ is the prior state estimate, representing the predicted result for the nodule in the current slice. ${\hat{x}}_{k-1}^{-}$ is the posterior state estimate from the previous slice, denoting the optimal result obtained. $u_{k-1}$ is the external control input vector. Since pulmonary nodules are not subject to external control, $u_{k-1}$ is set as a 4×1 zero vector. *A* is the state transition matrix. It is set as a 4×4 identity matrix. This choice assumes zero inter-slice displacement of the nodule center, which is consistent with the static nature of pulmonary nodules as anatomical structures. The apparent positional variation across consecutive slices results primarily from differences in imaging geometry rather than physical motion. This residual variation is captured by the process noise covariance *Q*, rather than being explicitly modeled in the state transition.

(13) $$P_{k}^{-}=AP_{k-1}A^{T}+Q$$

where $P_{k}^{-}$ is the error covariance matrix of the prior state estimate, quantifying the covariance between the predicted and actual results in the current slice. $P_{k-1}$ is the error covariance matrix of the posterior state estimate from the previous slice, representing the covariance between the actual and optimal results in that slice. *Q* is the process noise covariance matrix. It is set as a 4×4 identity matrix, assuming independent and identically distributed Gaussian noise across the state dimensions. This choice is justified by the minimal displacement of pulmonary nodules between adjacent slices.

The update step integrates the prior state estimate (the prediction) with the raw detection output $D_{k}$. This fusion outputs the posterior state estimate ${\hat{x}}_{k}$. The process is defined by (14)–(16).

(14) $$K_{k}=P_{k}^{-}H^{T}{\left(HP_{k}^{-}H^{T}+R\right)}^{-1}$$

where $K_{k}$ denotes the Kalman gain matrix, ranging from 0 to 1, which weights the prediction and the observation. A larger $K_{k}$ value indicates greater confidence in the observation. *H* is the observation matrix, set as a 4×4 identity matrix. *R* is the observation noise covariance matrix. It is set as a 4×4 identity matrix. This reflects uniform measurement uncertainty across the bounding box parameters.

(15) $${\hat{x}}_{k}={\hat{x}}_{k}^{-}+K_{k}\left(z_{k}-H{\hat{x}}_{k}^{-}\right)$$

where ${\hat{x}}_{k}$ is the posterior state estimate, obtained by fusing the prediction and the observation. It represents the optimal estimate of the nodule in the current slice. $z_{k}$ is the observation vector, derived from the conversion of $D_{k}$. *I* denotes the identity matrix.

(16) $$P_{k}=\left(I-K_{k}H\right)P_{k}^{-}$$

where $P_{k}$ denotes the error covariance matrix of the updated posterior state estimate.

The pseudocode of the CNT algorithm is shown in Algorithm 1. It computes the Euclidean distance between the center of each detection and each tracker’s predicted position. If this distance falls below a threshold, the detection matches the tracker. The algorithm then updates the matched tracker’s state and marks its tracking queue. If a detection matches no tracker, the algorithm initializes a new tracker. Finally, the algorithm uses Kalman filter predictions to fill missed detections for trackers with unmatched queue flags. This process completes the tracking cycle.

**Figure 7:**
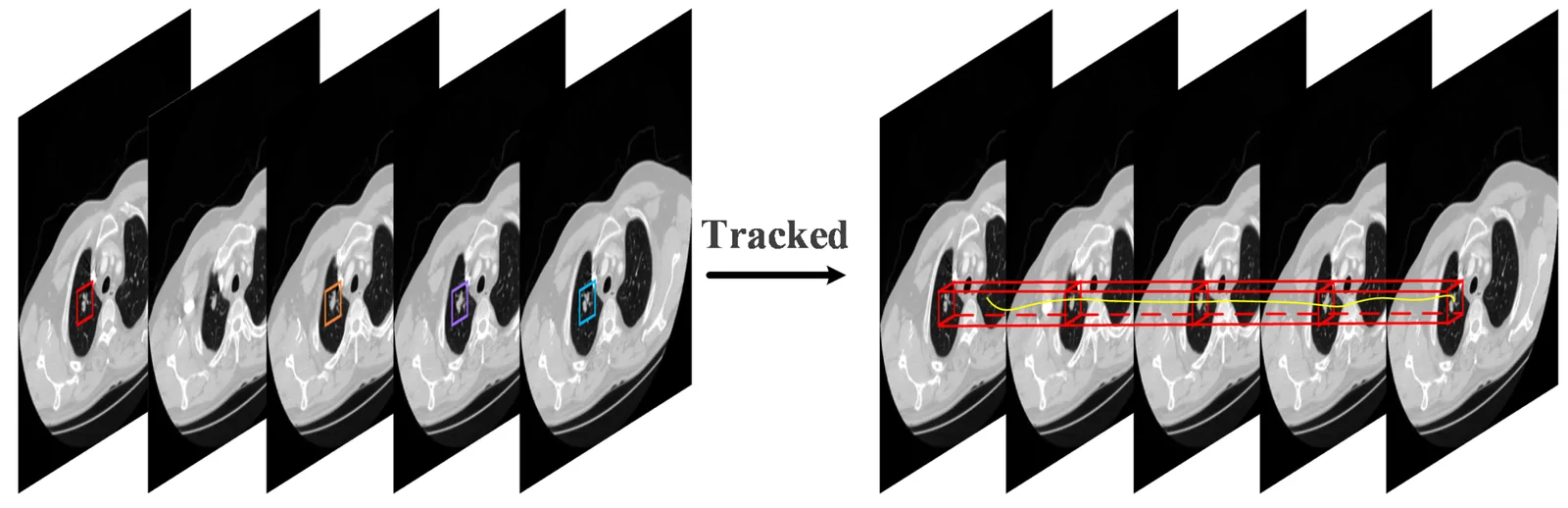
The tracking aggregation results after applying the CNT algorithm. The left part of the figure is a sequence of slices with detection boxes. The right part is the aggregation results with tracking. The red box represents the aggregated position, and the yellow lines represent the tracking trajectory of the pulmonary nodule.

Each successfully tracked nodule outputs a continuous state trajectory that spans multiple slices, which is both smooth and free of redundancies, as shown in Figure 7.
**Algorithm 1** Candidate Nodules Tracking**Input:** Ordered CT slices $I=\{I_{1},I_{2},...,I_{N}\}$. For each slice $I_{i},i\in \left[1,N\right]$, the detection network outputs a set of bounding boxes $D_{i}=\left\{D_{i,1},D_{i,2},...,D_{i,N}\right\}$. The set of observation vector $Z_{j}=\left\{z_{j,1},z_{j,2},...,z_{j,N}\right\}$, reshaped from $D_{k}$ with confidence coefficient. θ: distance threshold for centroid association (set to 5 pixels).  **Output:** The set of aggregated slices $T=\{T_{1},T_{2},...,T_{m}\}$. The prediction with Tracker $T_{i}=\left\{t_{1},t_{2},...,t_{n}\right\}$. Tracked queue list $Q=\{q_{1},q_{2},...,q_{l},q\in \left\{0,1\right\}\}$ of each tracker to judge whether the tracker is matched.    1:**for **i=1,2,...,N** do**  2:    **for** k=1,2,...,m **do**  3:        **for** $z_{j}\in Z_{j}$ **do**  4:            Compute the Euclidean distance *d* between the center of detection $z_{j}$ and the predicted position of tracker $T_{k}$;  5:            **if** d<θ **then**  6:                Update the matched trackers $T_{k}$ with matched detection $z_{j}$;  7:                $T_{k}.Q\left[-1\right]\leftarrow 1$;  8:            **else**  9:                Initialize a new tracker $T_{k+1}$ for unmatched detection $z_{j}$;10:                $T_{k+1}.Q\left[-1\right]\leftarrow 1$;11:                $T_{k}.Q\left[-1\right]\leftarrow 0$;12:            **end if**13:        **end for**14:        Fill the missing detections for CT slice where $T_{k}.Q\left[-1\right]=0$ by Kalman filtering;15:        $T_{k}.Q\left[-1\right]\leftarrow 1$;16:    **end for**17:**end for**

### 2.4. Stage II: False Positive Reduction

The first stage outputs a set of candidate nodules. At the level of 2D slices, structures such as vessel cross-sections and bronchial bifurcations remain highly similar to pulmonary nodules in terms of appearance features. Stage II aims to perform discrimination on the candidate set by introducing spatial morphological information.

**Figure 8:**
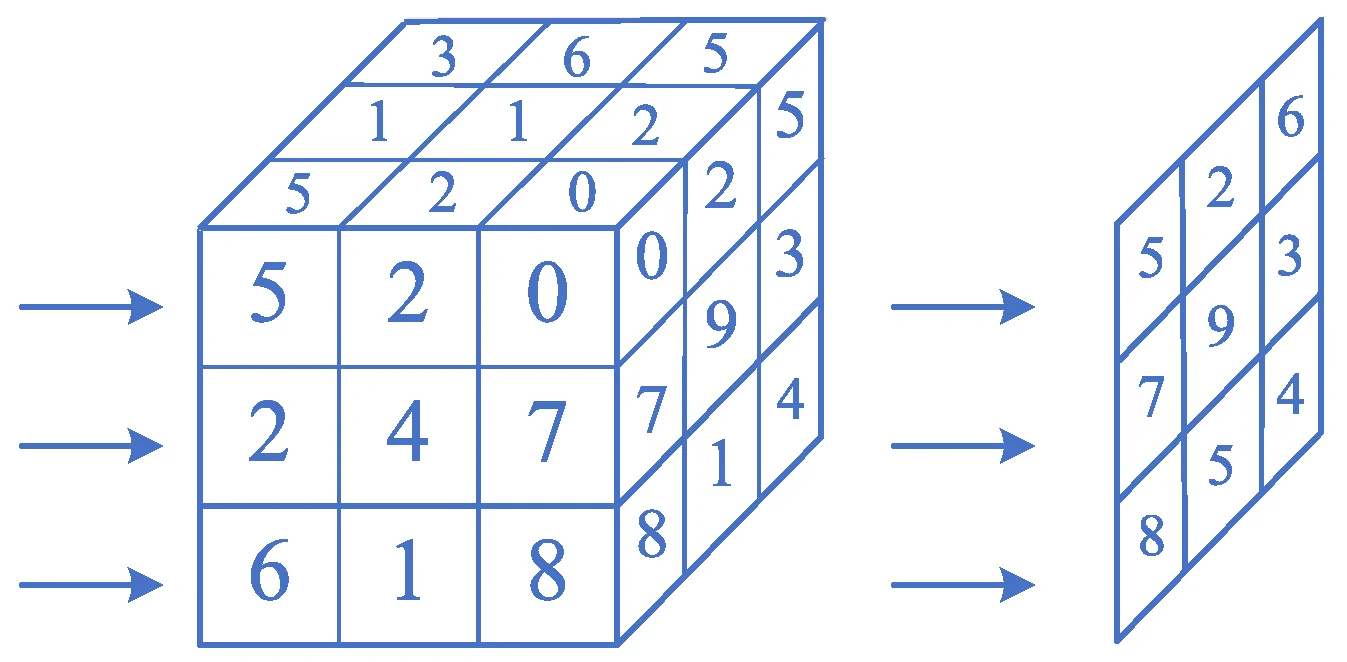
An example for MIP. The arrow indicates the direction of projection. The left cube consists of 3 three-dimensional 3 × 3 arrays. The plane on the right is the projection result of MIP.

Stage II designs a false positive filter module based on cross-slice Maximum Intensity Projection (MIP). MIP is a commonly used visualization method in medical image processing, as an example illustrated in Figure 8. The MIP selects the maximum intensity value along the line of sight through a volumetric dataset from a specific viewing angle. Then it projects the maximum intensity value into a two-dimensional plane. The maximum intensity value typically refers to the pixel with the highest brightness or density. This process generates a two-dimensional image that represents the most prominent features.

The method of Zheng et al. [34] employs MIP as a pre-processing step prior to the detection network. Unlike their approach, our work applies MIP to candidate nodules identified from the first stage. These candidates possess defined 3D spatial boundaries. For each set of candidate nodules determined by the CNT algorithm, a core sequence of $N^{\prime }=3$ slices is first identified. This core sequence is symmetrically extended by k=2 auxiliary slices (one on each side), yielding a total of 5 slices. MIP is then performed along the scanning axis on this extended sequence.

**Figure 9:**
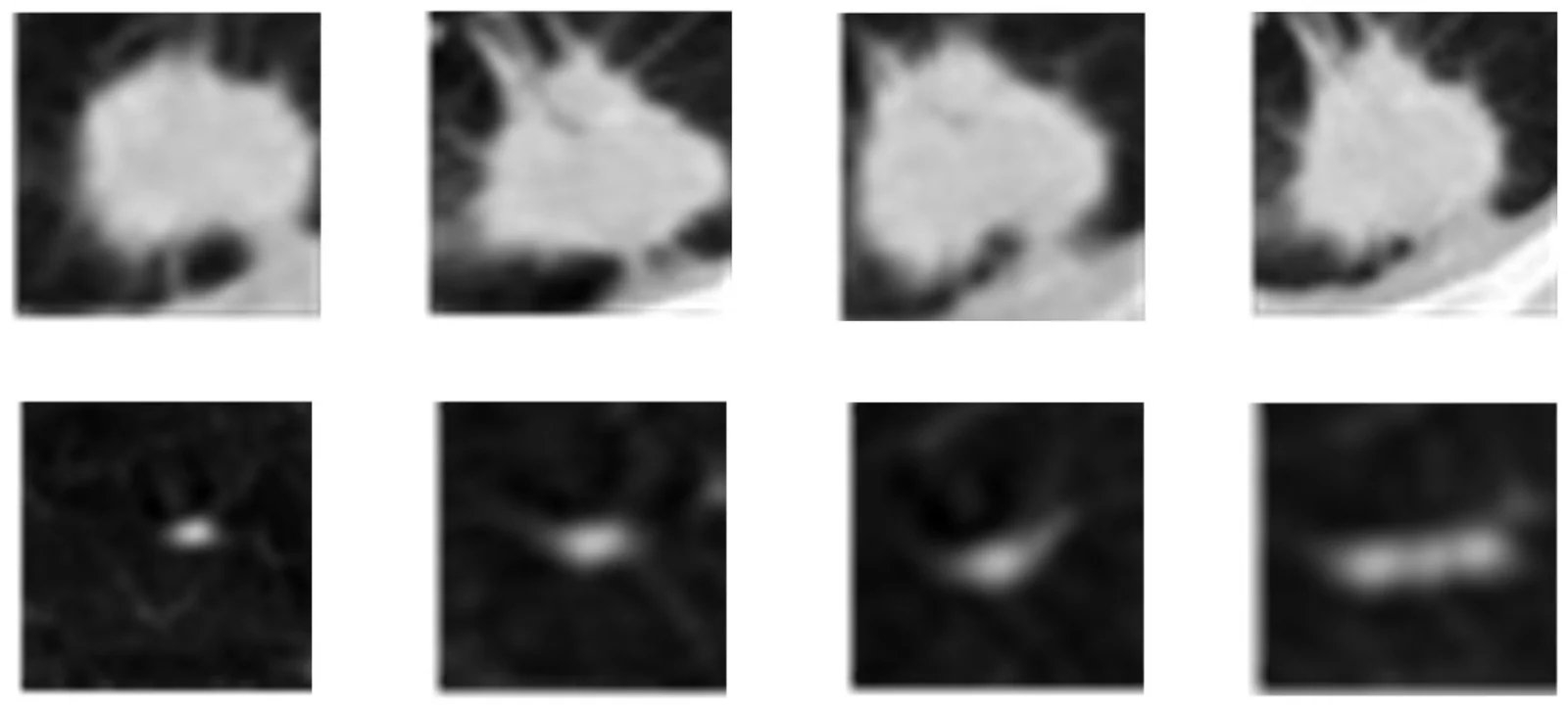
Projection effect of pulmonary nodules and blood vessel imaging. The first row shows the projection images of four different nodules. The second row shows the projection images of four different blood vessels. All projection images are displayed at a resolution of 224×224 pixels, which is the input resolution of the classification network used in Stage II.

This operation compresses the spatial information of the candidate nodule into a single 2D feature image. Nodules that approximate a sphere are projected into dense, quasi-circular bright spots, while tubular blood vessels appear as elongated stripes or bifurcated structures. This process highlights the inherent morphological differences, as shown in Figure 9. Each candidate region output from Stage I is cropped from the CT scan based on its bounding box coordinates. The cropped region is then resized to a fixed resolution of 224×224 pixels to serve as input to the subsequent binary classification network.

### 2.5. Stage II: 2D Classification Model

The generated MIP images are fed into a lightweight 2D CNN for binary classification of true vs. false nodules. The network is constructed based on the ResNet-50 architecture, which consists of 50 convolutional and fully connected layers with approximately 25.6 million trainable parameters. It accepts an MIP image of size 224×224×3 as input. ResNet-50 employs residual connections to mitigate the vanishing gradient problem. The core residual blocks employ a dual-pathway design. One pathway executes a series of convolutional operations, while the other functions as an identity skip connection. The outputs from both pathways are summed and then processed by the activation function ReLU. The features pass through a global average pooling layer and a fully connected layer. These layers produce the binary classification output for probabilities.

**Figure 10:**
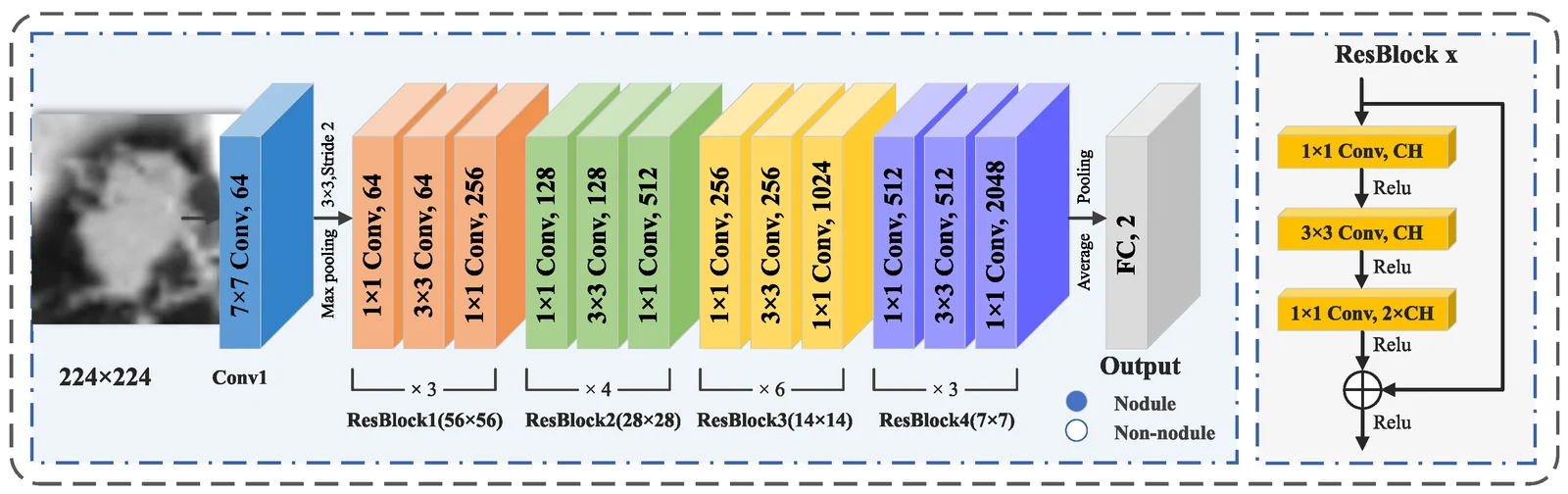
The classification network architecture of False Positive Reduction. Colors are used solely to distinguish different modules within this figure.

Figure 10 illustrates the architecture of the pulmonary nodule false positive reduction network built upon ResNet-50 in this study. The input is a candidate nodule image of size 224×224×3. This input is processed sequentially by a standard convolutional layer, a max-pooling layer, and three residual blocks. This outputs a feature map with dimensions of 7×7×2048. The feature map undergoes global pooling and a fully connected layer, resulting in a two-dimensional vector. This vector contains the classification probabilities for the two classes: Nodule and Non-Nodule. The task in this paper involves only two classes, and the training data has an equal number of samples for each class. The designed pulmonary nodule false positive reduction network employs the cross-entropy loss function. The formula is shown in (17).

(17) $$L=-\frac{1}{N}\Sigma_{1}^{N}\left[y_{i}\cdot \log\left(\bar{y_{i}}\right)+\left(1-y_{i}\right)\cdot \log\left(1-\bar{y_{i}}\right)\right]$$

where *N* denotes the total number of samples, $y_{i}$ represents the true label of the *i-th* sample. $\bar{y_{i}}$ denotes the predicted probability for the *i-th* sample.

The guiding principle of this module is to prioritize the high sensitivity required in clinical screening and strictly control missed diagnoses. Hence, a conservative filtration strategy is adopted. A candidate is excluded only if the classification network identifies it as a false positive with extremely high confidence. Otherwise, the candidate nodule is retained as a reliable detection result.

## 3. Experimental Setup

### 3.1. Datasets

The Lung Nodule Analysis 2016 (LUNA16) [45] dataset is used for experiments and evaluation in this paper, which is a subset of the LIDC-IDRI [46] dataset. LIDC-IDRI is a public dataset for lung tumor detection and diagnosis, jointly created by radiologists and computer scientists from the National Institutes of Health (NIH) in the United States. The LUNA16 dataset contains 1018 CT scans from 1010 patients. The annotations of these scans were provided by 4 experienced thoracic radiologists in two phases. In the initial phase, all CT scans were independently reviewed by each radiologist, who categorized lesions as follows: “nodule ≥ 3 mm”, “nodule < 3 mm” and “non-nodule ≥ 3 mm”. In the subsequent phase, each radiologist’s own marks were reassessed alongside the anonymized marks of the other three radiologists to generate the final outcomes. LUNA16 removed CT scans with slice thickness greater than 2.5 mm and nodule diameter less than 3 mm, leaving 888 CT scans stored in (mhd/raw) format with 512×512 resolution per CT scan. In addition, the annotation information of 1186 nodules was provided in CSV file format.

In this study, we construct the experimental dataset by applying the LUNA16 filtering criteria directly to the LIDC-IDRI collection. Specifically, scans with slice thickness greater than 2.5 mm or nodule diameter less than 3 mm are excluded. Additional quality controls require each scan to have a complete CT slice sequence, valid annotation files without manual coordinate correction, and a strict one-to-one correspondence between images and annotation files. After filtering, the final dataset comprises 600 patients with 9741 CT slices and 3558 annotated bounding boxes, covering all 1186 nodules defined by LUNA16. The bounding box annotations are derived from the point-based nodule coordinates in the LUNA16 annotation files.

This study employs a five-fold cross-validation method to comprehensively evaluate the performance of the pipeline. According to the principle of random selection, each subset contains the training set, validation set and test set in an 8:1:1 ratio. The data split was performed at the patient level, ensuring that all slices from the same patient were assigned exclusively to a single fold.

For the false positive reduction classifier, training samples were generated by running the Stage I detector exclusively on the training split of each fold. Candidate regions with an IoU ≥0.2 with respect to ground-truth annotations were labeled as positive samples (nodules), the remainder were labeled as negative samples (non-nodules). The classifier was trained and evaluated strictly within the same fold, with no exposure to validation or test data at any stage. The training dataset for the classification model originates from the LUNA16 dataset, but differs from the official manually annotated false positive reduction labels. False positive nodules refer to other tissues that are difficult to distinguish in the detection model, which are highly similar to pulmonary nodules in appearance and density, and still cannot be accurately differentiated at the algorithm level. Therefore, we obtained a large number of false positive pulmonary nodule samples in the LUNA16 dataset using a pre-trained pulmonary nodule detection model, and created the classification dataset in combination with the pulmonary nodule labels. The detection model used for false positive mining was trained exclusively on the training split of the same fold. It ensures that no validation or test data were involved in sample generation or classifier training.

### 3.2. Metrics

The performance of the pulmonary nodule detection algorithm is evaluated using the Free-Response Receiver Operating Characteristic (FROC) curve and the Coordinate-wise Performance Measure (CPM) score. The FROC curve is a curve used to evaluate the performance of algorithms for detecting multiple lesions in medical images. Its vertical axis represents the probability of detecting all pulmonary nodules in each image, as shown in (18), expressed as sensitivity. The horizontal axis represents the number of false positive pulmonary nodules detected in each image, as shown in (19), expressed as Average FPs per scan.

(18) $$Sensitivity=\frac{TP}{TP+FN}$$

(19) $$FPs/scan=\frac{Total\;FPs}{Total\;Scans}$$

where TP (true positives) represents the number of samples correctly predicted as pulmonary nodules. FN (false negatives) represents the number of samples incorrectly predicted as non-nodules. Total FPs (total false positives) represents the total number of samples incorrectly predicted as pulmonary nodules. Total Scans represents the total number of scanned images.

The CPM score represents the mean sensitivity at Average FPs per scan of (0.125, 0.25, 0.5, 1, 2, 4, 8), as shown in (20).

(20) $$CPM=\frac{1}{N}\sum_{i=\{0.125,0.25,0.5,1,2,4,8\}}Sensitivity_{FPs/scan=i}$$

The value of *N* is 7 and $Sensitivity_{FPs/scan=i}$ represents the sensitivity value at *i* false positives per scan.

In addition to the FROC-based metrics, we report Precision, Recall, and F1-score to provide a complementary assessment of detection quality. These metrics are defined as:

(21) $$Precision=\frac{TP}{TP+FP}$$

(22) $$Recall=\frac{TP}{TP+FN}$$

(23) $$F1=2\cdot \frac{Precision\cdot Recall}{Precision+Recall}$$

where TP, FP, and FN denote the numbers of true positives, false positives, and false negatives, respectively. These metrics are computed at each operating point on the FROC curve and are reported alongside the CPM score.

### 3.3. Implementation Details

The algorithms in this study were implemented on the PyTorch 1.7.0 framework, with Python 3.7. Model training was performed on an NVIDIA GeForce RTX 3060 GPU (NVIDIA Corporation, Santa Clara, CA, USA) with 12 GB memory. CUDA 11.4 tools were utilized to improve training speed. On the NVIDIA GeForce RTX 3060 GPU, the improved YOLOX detector processes a single 512×512 CT slice in 58.27 ms on average, corresponding to approximately 17.16 FPS. For the detection model, the stochastic gradient descent (SGD) optimizer was adopted with a momentum of 0.937 and a weight decay of $5\times 10^{-4}$. The initial learning rate was set to 0.01, with a cosine annealing schedule. A two-stage training strategy was employed: the backbone was frozen for the first 50 epochs with a batch size of 8, followed by 200 epochs of full-parameter training with a batch size of 4, totaling 250 epochs. For the false positive reduction classifier, the Adam optimizer was used with an initial learning rate of $10^{-4}$ and a batch size of 8, trained for 100 epochs.

Data augmentation was applied during training to improve model generalization. Mosaic augmentation, which stitches four randomly sampled images into one, was applied with a probability of 0.5 and was only active during the first 70% of training epochs. MixUp, which blends two images linearly, was applied with a probability of 0.5. Color jitter was employed in the HSV space with hue factor 0.1, saturation factor 0.7, and value factor 0.4. Geometric transformations included random horizontal flipping, random scaling and distortion, and random rotation within ±15 degrees. Letterbox padding was used to preserve the aspect ratio when resizing images to the required input dimensions.

## 4. Results

### 4.1. Performance of the Enhanced 2D Detector

To evaluate the contribution of the proposed components to detection performance, we conducted an ablation study. As shown in Table 1, A, B, C, and D represent four Configurations involved in the ablation experiment. Config A denotes the baseline YOLOX network model. Config B adds the feature fusion module (WFF) to the baseline YOLOX. Config C incorporates both the WFF module and the GIoU loss into the baseline. Config D integrates the WFF module, the GIoU loss, and the NWD loss into the baseline model. The table presents the sensitivity of these four configurations at 1 false positive per scan (FPs = 1). The three components contribute to varying degrees of improvement in pulmonary nodule detection accuracy. These configurations (A–D) are used throughout the ablation study section.

**Figure 11:**
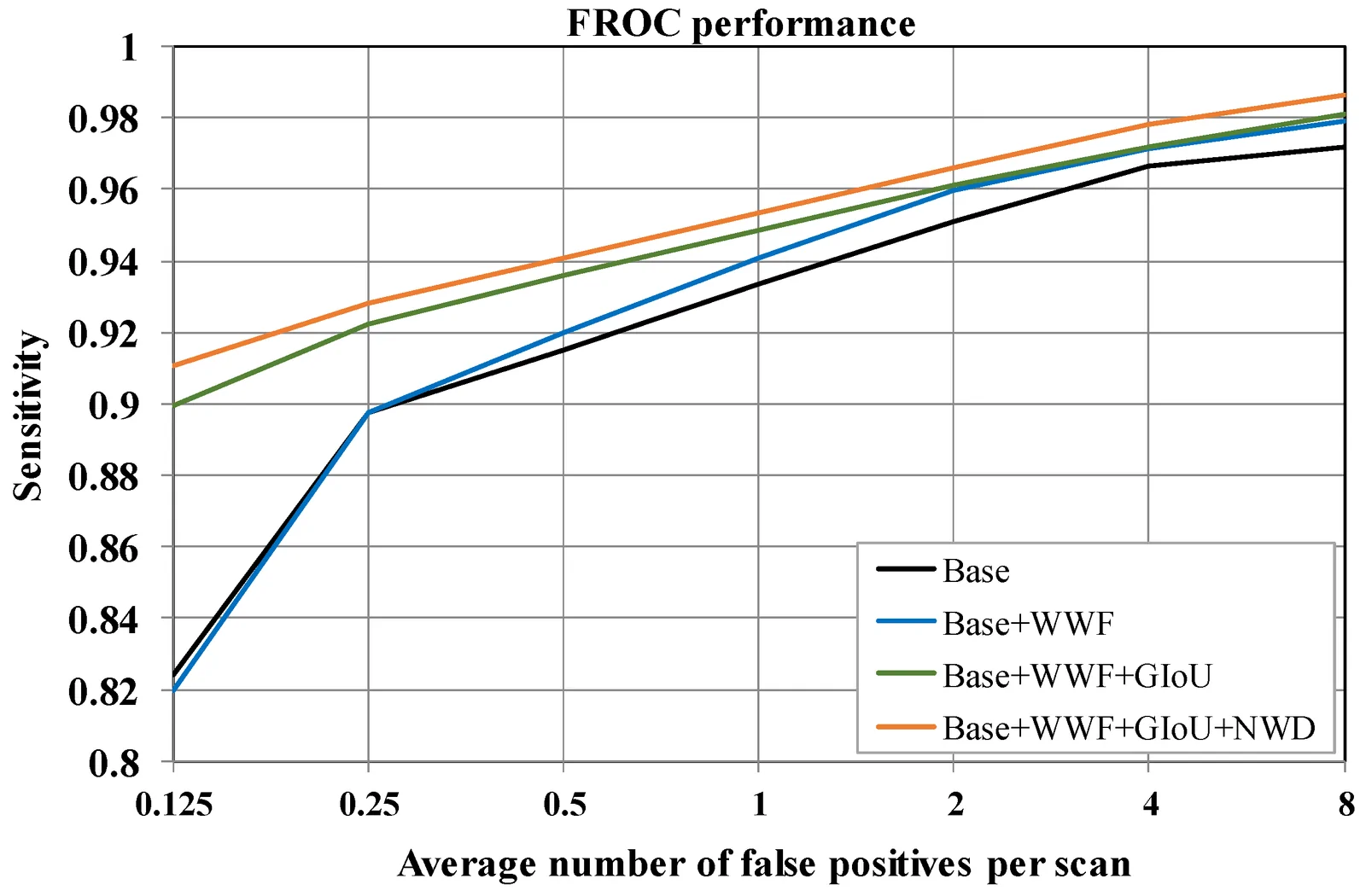
FROC curves of different model configurations in the ablation study on the LUNA16 dataset.

As shown in Figure 11, we compare the detection performance of four model configurations on the LUNA16 dataset, obtaining the FROC curves. The complete model (Config D) achieves the highest sensitivity across all false positive levels (0.125 to 8.0 FPs/scan). It shows that the proposed improvement modules have initial effectiveness.

**Figure 12:**
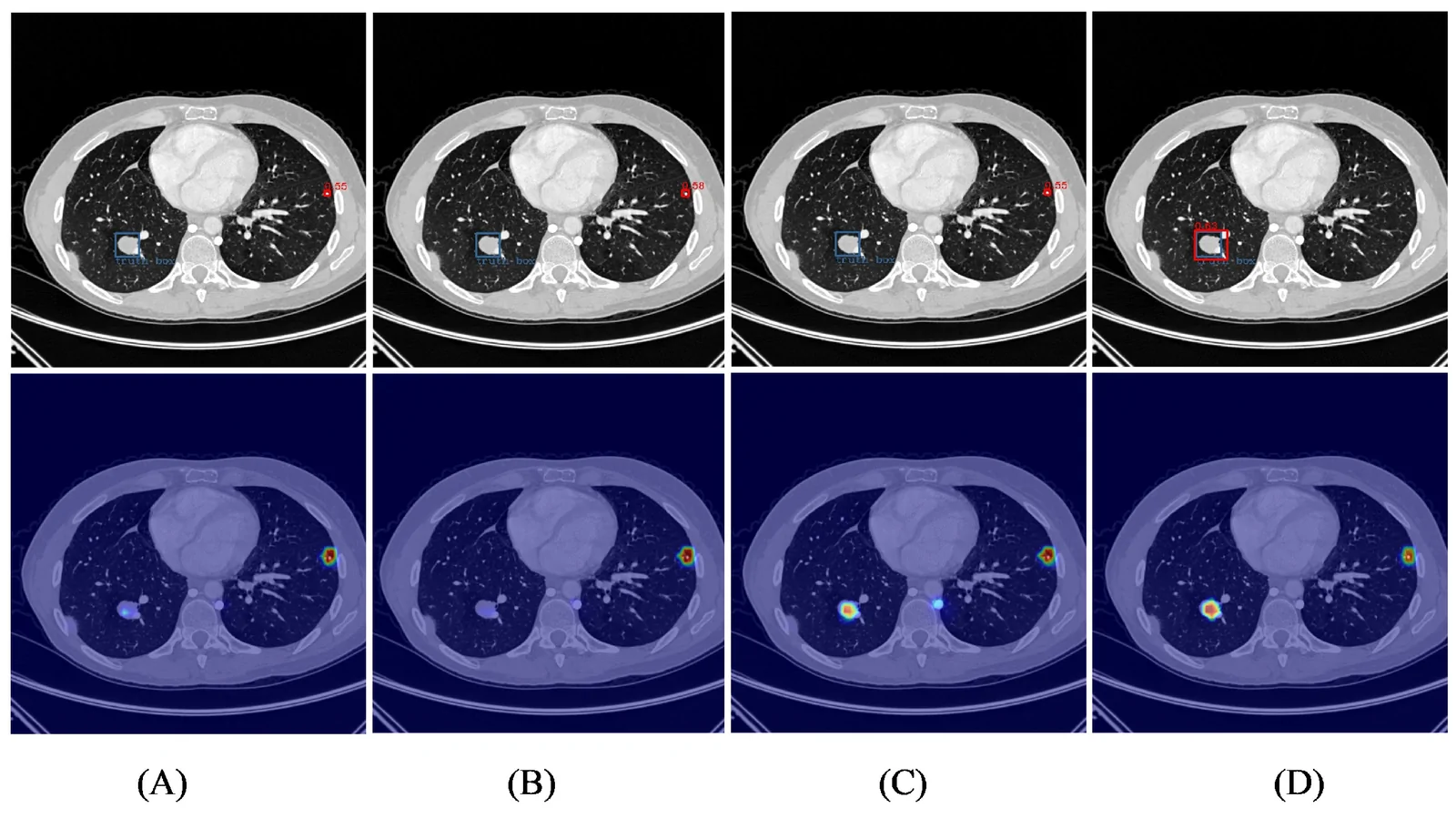
Visual comparison of detection results and corresponding class activation heatmaps for the four model configurations. The top row shows CT slices with ground-truth nodules (blue box) and model-predicted bounding boxes (red box). The bottom row displays the class activation heatmaps for the corresponding slice, where the pseudo-color intensity represents the model’s confidence level (warmer colors indicate higher confidence). Columns (**A**–**D**) correspond to Config A, B, C, and D.

Figure 12 shows the comparison of detection results for each configuration and class activation heatmaps on a representative CT slice. In the baseline model (Config A), the high-confidence activation region is concentrated in the upper-right corner of the image. This region corresponds to a false positive structure that resembles a nodule. In contrast, the response to the actual nodule remains weak. With the addition of the WFF module (Config B), this situation shows no fundamental change. Following the introduction of the GIoU loss (Config C), although the predicted bounding box remains unadjusted, the heatmap reveals an enhancement in the activation signal within the true nodule area. The complete model (Config D) successfully directs both the strongest activation signal and the prediction box to accurately lock onto the true nodule location, while it effectively reduces the response to the false positive target. This visual evidence objectively demonstrates how the improvement modules progressively optimize the model’s feature focus and localization capability.

### 4.2. Effectiveness of Candidate Nodule Tracking

We introduced the Candidate Nodule Tracking (CNT) algorithm based on Kalman filter, to show how to utilize the spatiotemporal information between consecutive CT slices. This subsection studies the impact of the tracking window size ($N^{\prime }$) on system performance. The parameter $N^{\prime }$ defines the number of consecutive slices processed in a single association step by the algorithm. Table 2 shows the comparison of system performance under different *N*′ values. When setting $N^{\prime }=3$, the system outputs the highest CPM score (0.9218) and the best sensitivity across all predefined false positive levels (0.125–8 FPs/scan). Increasing the window size to $N^{\prime }=5$ and $N^{\prime }=7$ reduces the CPM to 0.9049 and 0.9041. These results show that a large window does not provide additional performance gains. It can lead to a slight performance degradation. This clearly identifies $N^{\prime }=3$ as the optimal choice under our experimental setup.

**Figure 13:**
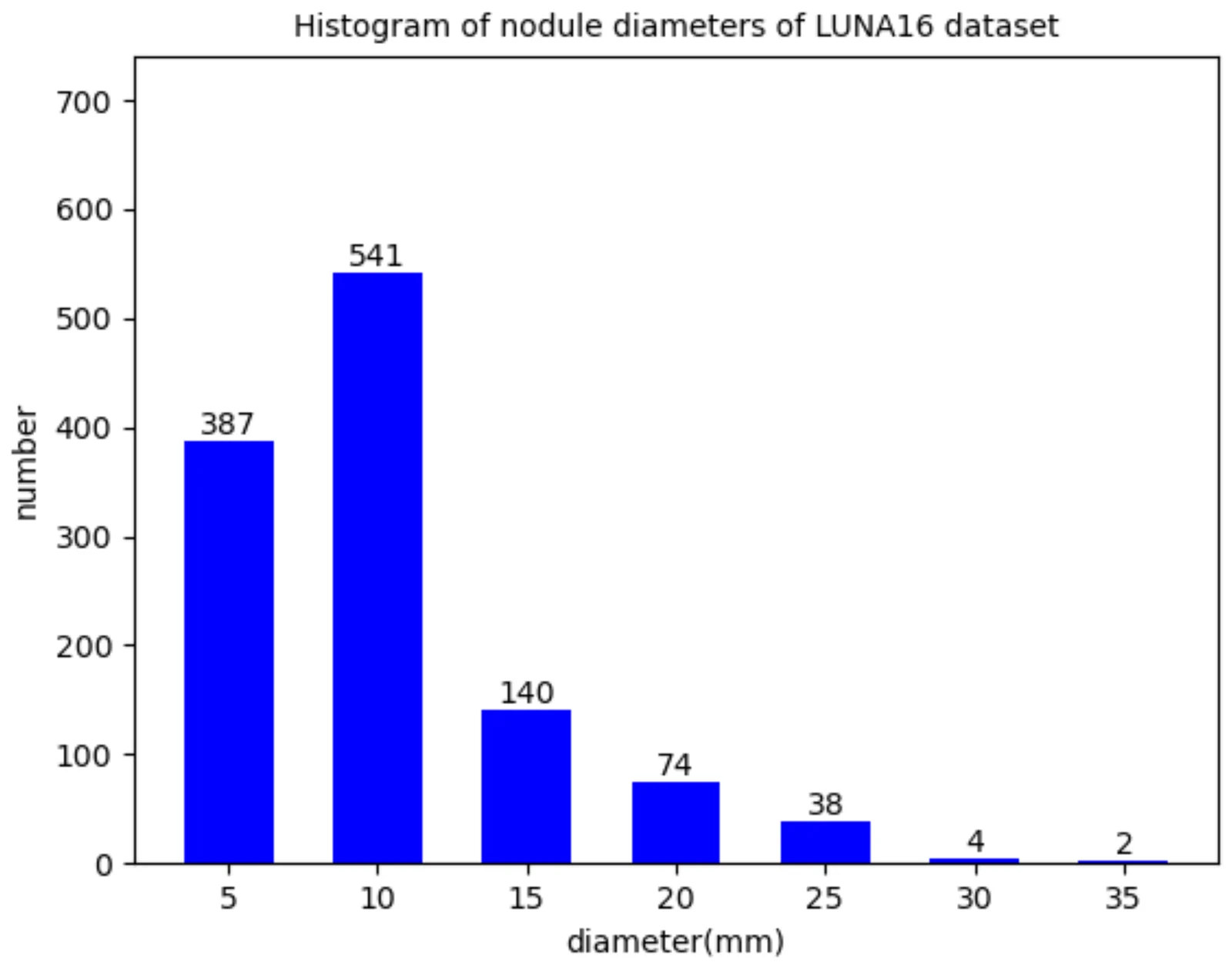
Histogram of nodule diameters of LUNA16 dataset.

To understand the aforementioned performance trend and provide context, we examine the physical characteristics of the nodules in the LUNA16 dataset. Figure 13 presents a histogram of the diameter distribution for annotated nodules in the dataset. The majority of nodules have diameters concentrated within the 5–10 mm range. Given the slice thickness of the dataset, nodules within this diameter range are typically visible across 2 to 4 consecutive slices. This observed distribution aligns with the choice of $N^{\prime }=3$.

### 4.3. Influence of Auxiliary Slice Number on MIP-Based Classification

**Figure 14:**
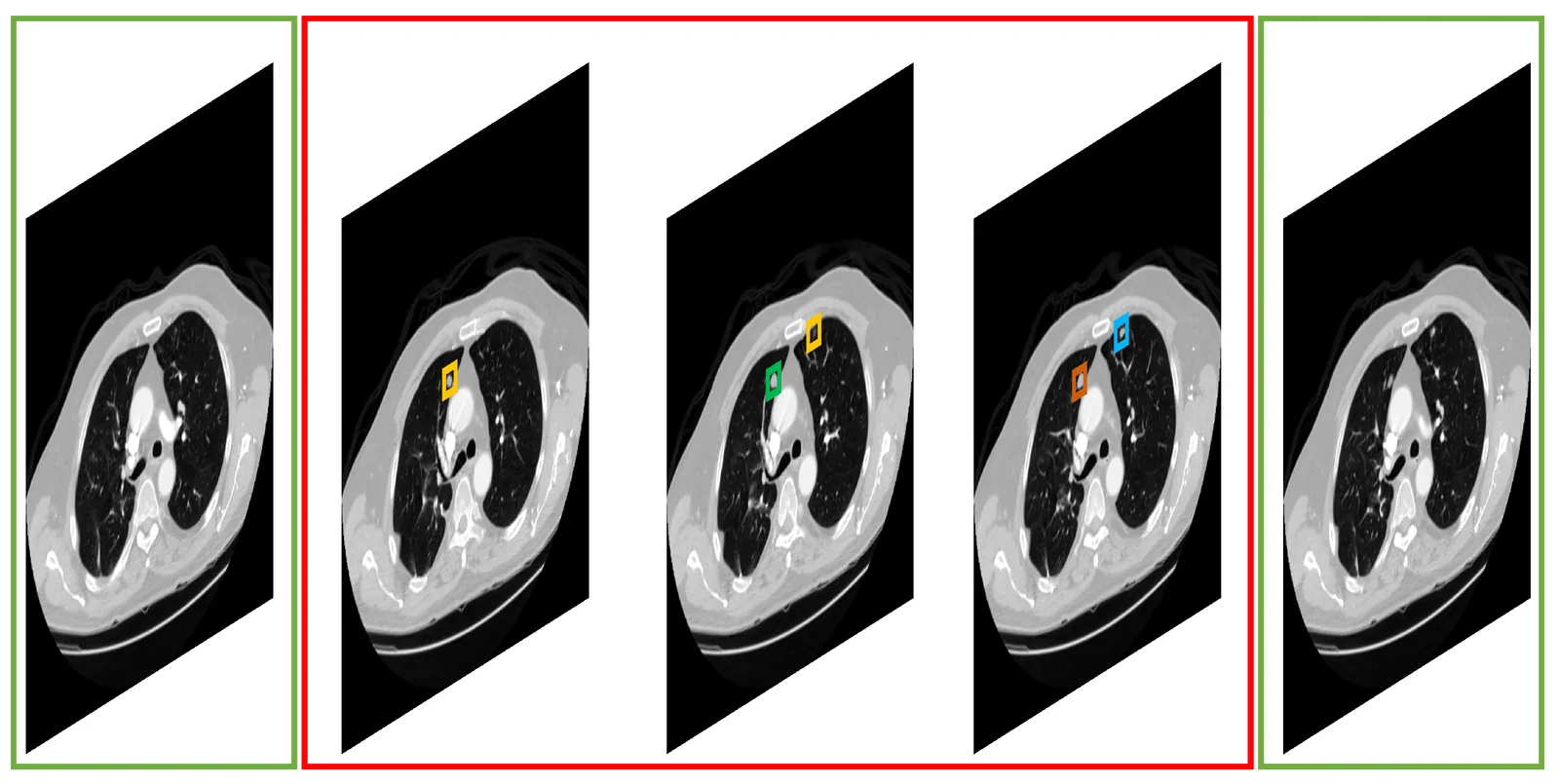
An example of placement for auxiliary slices. The red boxes represent the fused results, while the green boxes represent slices providing spatial auxiliary information. The small colored boxes represent nodule candidate regions detected by the Stage I detector across consecutive slices. Different colors distinguish different candidate boxes.

In the second stage for false positive reduction, we employ cross-slice Maximum Intensity Projection (MIP) to enhance the morphological features of the candidate regions. As shown in Figure 14, the red boxes represent the core slice sequence for each candidate nodule, determined by the CNT algorithm. The example shown uses a sequence of 3 slices. To investigate the influence of spatial information, we symmetrically extend this core sequence by k adjacent slices on both ends. The added slices serve as auxiliary information, indicated by the green boxes in the figure. MIP is subsequently performed along the slice-stacking direction, generating a single two-dimensional feature map.

**Figure 15:**
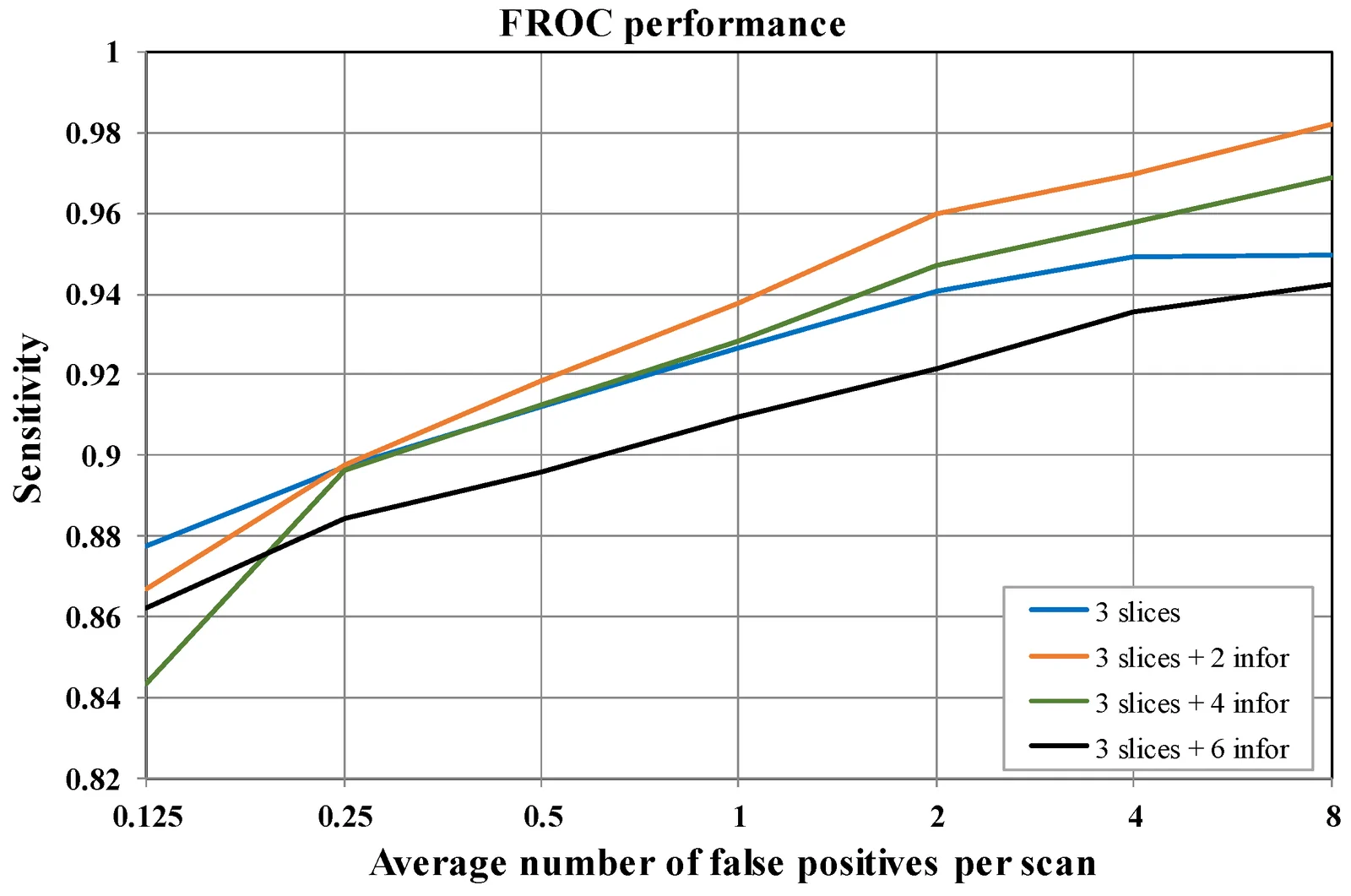
The comparison of detection performance in pulmonary nodule detection after false positive reduction with the application of different numbers of auxiliary slices.

We evaluate the impact of the number of auxiliary slices k on the final classification performance. Figure 15 shows the FROC curves of corresponding false positive reduction performance for k=0,2,4,6. The experimental results indicate that introducing auxiliary slices improves classification discriminability. When k=2, the system achieves the highest sensitivity at major false positive levels. Increasing the auxiliary information to k=4 results in a plateauing or slightly fluctuating performance gain. When k=6, an observable decline in sensitivity occurs across all operating points. This suggests that an excessive amount of auxiliary information introduces interference to the classification process.

**Figure 16:**
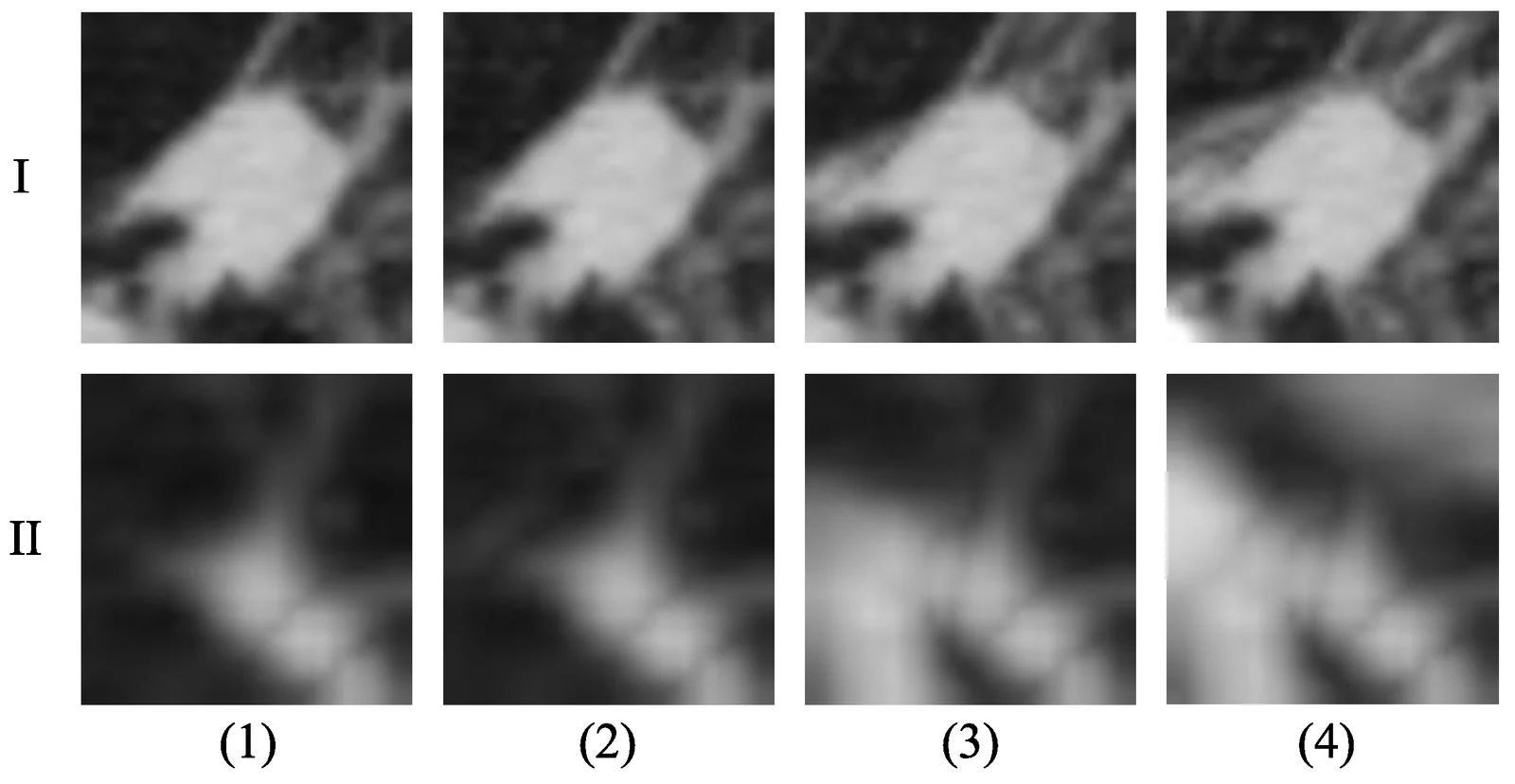
The projection images of nodule (**I**) and non-nodule (**II**) using different numbers of auxiliary slices. (**1**) 3 slices, (**2**) 3 slices + 2 auxiliary slices, (**3**) 3 slices + 4 auxiliary slices, (**4**) 3 slices + 6 auxiliary slices.

The visual differences in MIP images under different configurations provide an intuitive explanation for the aforementioned performance trends. Figure 16 compares the projection results of the same true nodule (Row I) and a typical blood vessel for false positive (Row II) under different k values. When k=0, the projection originates solely from the core sequence. The two-dimensional morphological distinction between the nodule and the vessel is limited at this stage. With k=2, the nodule’s projection appears as a denser, more uniform quasi-circular bright spot. In contrast, the vessel manifests as an elongated or irregular stripe. The visual contrast between the two is most pronounced in this case. As the *k* value increases to 4 and then 6, the projections of both structures become more diffuse. The overall image grayscale tends to homogenize, resulting in blurred feature boundaries and weakened distinctiveness. This visual change objectively shows that adding a few auxiliary slices is helpful, while adding too many becomes harmful for reduction.

To evaluate the contribution of each stage in the pipeline, we conducted a module-level ablation study. Table 3 presents the FROC sensitivity at seven standard false positive levels and the corresponding CPM score for three configurations: the improved detector alone, the detector with Kalman tracking, and the complete two-stage framework. The complete framework achieves the highest CPM of 0.934, with consistent improvements across all false positive levels from 0.25 to 8.0 FPs/scan, demonstrating the complementary effectiveness of each module.

A stratified analysis of the CPM scores further reveals the performance characteristics of each configuration across different false positive regimes. For the complete framework, the CPM reaches 0.901 in the low false positive region (0.125–0.5 FPs/scan), 0.948 in the medium region (1.0–2.0 FPs/scan), and 0.972 in the high region (4.0–8.0 FPs/scan). The substantial improvement from the low to medium region indicates that the framework achieves near-maximal sensitivity at clinically acceptable false positive rates, while the high-region CPM of 0.972 confirms that the tracking and classification modules effectively recover missed detections and suppress false positives when the detection threshold is relaxed.

### 4.4. Overall System Performance and Comparison with State-of-the-Art

As shown in Table 4, we compare our method with other mainstream approaches on the LUNA16 dataset. These compared methods encompass different technical approaches, including strategies based on 2D multi-view MIP, cascaded pipeline using 3D segmentation and classification, and hybrid 2D/3D networks. Our method achieves the highest overall Competition Performance Metric (CPM) score of 0.9344. It demonstrates leading sensitivity at 2.0, 4.0, and 8.0 false positives per scan (FPs/scan). The proposed pipeline shows competitive overall performance.

At the clinically relevant operating point of three candidate boxes per scan with a confidence threshold of 0.1, the complete framework achieves a Recall of 0.9316, a Precision of 0.7543, and an F1-score of 0.8330. The high Recall reflects the framework’s effectiveness in detecting pulmonary nodules, while the Precision–Recall trade-off across different confidence thresholds is reported in Table 5.

## 5. Discussion

As shown in Table 1, the ablation study confirms that the WFF module, the GIoU loss, and the NWD loss synergistically improve detection performance through complementary mechanisms. The WFF module enhances the model’s attention to the critical features of nodules by performing weighted fusion of multi-scale features. When applied individually (Config B), it primarily improves detection sensitivity at medium-to-high false positive levels. The GIoU loss optimizes bounding box regression from the perspective of global geometric overlap. The NWD loss increases localization sensitivity for small and ambiguous targets. Together, the GIoU and NWD losses address the precise regression requirements for samples.

The progressive changes in the heatmaps (Figure 12) visually confirm this synergistic mechanism. In the baseline model (Config A), the attention is dispersed across false positive regions. The introduction of the GIoU loss (Config C) enhances the activation signal in the true nodule area. The integration of the NWD loss (Config D) locks the model’s attention onto the true nodule. These three modules progressively optimize the model’s feature selection and spatial localization capabilities. They enhance both detection accuracy and the interpretability of the model’s decisions.

This study explicitly models the spatial continuity of nodules across consecutive slices within a single CT scan. Experiments determined that the optimal tracking window size for the CNT algorithm is $N^{\prime }=3$. This optimal window size corresponds to the observed fact that most nodules in the LUNA16 dataset appear within 2 to 4 consecutive slices (Figure 13). The alignment between the window size and the nodule diameter distribution supports the validity of the tracking design. The window is large enough to capture the full extent of most nodules, yet small enough to limit the inclusion of unrelated slices that would increase false positives.

This detection-tracking co-design offers two advantages. First, it enables proactive recovery of missed detections. When a nodule is missed on individual slices by the 2D detector, the CNT algorithm predicts its position from adjacent slices and fills the gap. This design can decrease missed diagnoses at the source. Second, it achieves automatic aggregation of redundant proposals. The system consolidates multiple detection boxes from different slices belonging to the same nodule into a unified candidate, reducing redundancy. Our co-design simulates the sequential slice-reviewing cognition of doctors and embeds cross-slice reasoning into the detection pipeline.

Existing pulmonary nodule detection methods lack the architectural capability for missed-detection recovery. Three-dimensional end-to-end approaches process the entire CT volume and output results in a single forward pass. Slice-wise tracking has no operational premise in this paradigm. Two-dimensional consecutive slice methods are built upon slice-wise independent detection. Their two-stage frameworks suffer from two structural limitations. First, several methods pass candidate location points or segmentation masks between stages. They discard the detection box sequence and thus lose spatial information. Second, methods that retain detection boxes optimize only single-frame accuracy in Stage I, with insufficient sensitivity to center-point offsets for small targets. Our experiments show that when detection boxes are imprecisely localized or inappropriately sized, cross-slice association cannot be established and tracking subsequently breaks down. Our missed-detection recovery approach is therefore used jointly with the improved Stage I detector. This detector enhances localization accuracy through the GIoU loss and constrains inter-slice smoothness through the NWD loss. The ablation results in Table 3 confirm that the detector improvements and the tracking mechanism constitute an interdependent co-design. Neither module alone is sufficient to achieve the full missed-detection recovery capability. The effectiveness of integrating detection and tracking has also been demonstrated in other medical image analysis tasks [54].

The FROC comparison in Table 4 reveals that at the very low false positive level (0.125 FPs/scan), our sensitivity (0.865) is slightly below that of Zhang et al. (0.892) [52]. We attribute this to the inherent cost of slice expansion: the CNT algorithm extends the core sequence by auxiliary slices, some of which may not contain nodules. FROC evaluation is slice-level. These slices are counted as false positives when the output quota is extremely limited. Some extended slices occupy the available slots and lower the sensitivity.

Low performance at the very low false positive level is also confirmed by the module-level ablation in Table 3. After adding the MIP-based false positive reduction stage, the sensitivity at 0.125 FPs/scan drops from 0.878 (tracking-only) to 0.865. Since MIP takes the maximum intensity value, empty extended slices do not distort nodule morphology in the projection. The classifier cannot reliably identify and exclude them. At the very low false positive budget, these extended slices consume the available quota, causing the performance drop relative to the tracking-only configuration.

As shown in Table 4, when the false positive constraint is relaxed, our method comprehensively surpasses all compared methods. The missed nodules recovered through slice expansion are now retained in large numbers, while the classifier effectively suppresses false positives without sacrificing these recoveries. In the clinically relevant range (2.0–8.0 FPs/scan), the sensitivities reach 0.955, 0.967, and 0.977, all ranking the highest among the compared methods.

The stratified CPM analysis in Table 3 further supports this increasing performance trend. The complete framework achieves CPM scores of 0.901, 0.948, and 0.972 in the low (0.125–0.5), medium (1.0–2.0), and high (4.0–8.0) false positive regions, respectively. This upward trend demonstrates that the overall benefit of slice expansion far exceeds its marginal cost at very low false positive budgets. The slice expansion enables both tracking-based missed-detection recovery and classifier-based false positive suppression to fully synergize.

In the false positive reduction stage, cross-slice MIP is employed to incorporate morphological features. The number of auxiliary slices, *k*, has an optimal value (k=2). An insufficient *k* value leads to inadequate information in the MIP. It results in limited morphological distinction between nodules and false positives such as blood vessels. An excessively large *k* value causes a degradation in performance. Visualization results confirm that too many slices lead to homogenized grayscale in the projected images, blurred feature boundaries, and reduced distinctiveness. This demonstrates that the MIP process involves a balance between enhancing morphological discriminability and introducing noise. Indiscriminately increasing information can, in fact, drown out the core features.

The complete framework proposed in this paper achieves competitive overall performance on the LUNA16 dataset, with a CPM of 0.9344. Its advantage is evident in the medium-to-high false positive regions (2.0–8.0 FPs/scan). This performance is primarily attributed to the synergistic design of the two-stage pipeline. The first stage reduces missed detections at their source through tracking. The second stage accurately discriminates these high-quality candidates using optimized morphological features. The pipeline demonstrates that incorporating interpretable components such as Kalman filtering and MIP can effectively balance high detection sensitivity with improved specificity.

Several limitations of this study should be acknowledged. The evaluation is conducted exclusively on the LIDC-IDRI dataset based on LUNA16 dataset standard. The generalizability of the proposed framework to diverse clinical populations, different CT acquisition protocols, and varying nodule characteristics remains to be validated on independent external datasets. The false positive reduction classifier was trained on candidate samples generated by the Stage I detector, which may introduce a dependency on the specific detection model. Future work will focus on validating the pipeline on multi-center cohorts and exploring domain adaptation techniques to enhance robustness across heterogeneous clinical settings.

## 6. Conclusions

This paper proposes a two-stage pipeline for accurate pulmonary nodule detection in CT scans. The core innovation lies in the detection-tracking co-design: the improved detector produces boxes of trackable quality, and the tracker exploits this quality to recover missed detections. The first stage integrates an enhanced YOLOX detector with a Kalman filter-based tracker to proactively recover missed detections and reduce redundancy. The second stage employs cross-slice MIP for false positive reduction using morphological features. Systematic ablation studies on the LUNA16 dataset demonstrate the effectiveness of each component and their synergy. The complete framework achieves a competitive CPM score of 0.9344, showing particular strength in medium-to-high sensitivity regions. This work provides a novel paradigm that balances high detection sensitivity with clinical interpretability for computer-aided diagnosis.

## Figures and Tables

**Table 1:** Ablation study of improved YOLOX network. The checkmark (✓) indicates that the corresponding module is included in the configuration.

Config	WFF	GIoU	NWD	Sensitivity (FPs = 1)
A				0.9333
B	✓			0.9406
C	✓	✓		0.9483
D	✓	✓	✓	0.9534

**Table 2:** The performance for detection results with different number of auxiliary slices. Bold values indicate the best performance.

Method	False Positive per Scan							CPM
	0.125	0.25	0.5	1.0	2.0	4.0	8.0	
$N^{\prime }=0$	0.7194	0.7541	0.7898	0.8261	0.8508	0.8740	0.8989	0.8162
$N^{\prime }=3$	**0.8776**	**0.8970**	**0.9121**	**0.9265**	**0.9408**	**0.9494**	**0.9495**	**0.9218**
$N^{\prime }=5$	0.8535	0.8729	0.8897	0.9059	0.9235	0.9393	0.9492	0.9049
$N^{\prime }=7$	0.8458	0.8652	0.8861	0.9090	0.9300	0.9450	0.9479	0.9041

**Table 3:** Module-level ablation study of the proposed framework. The detector in all three configurations is the improved YOLOX (Config D in Table 1, which integrates WFF, GIoU, and NWD). The three rows represent a progressive stacking of components. The first row is the detector alone. The second adds Kalman filter-based Candidate Nodule Tracking ($N^{\prime }=3$) based on config in row 1. The third further incorporates MIP-based false positive reduction with the ResNet-50 classifier based on config in row 2, forming the complete two-stage pipeline. The bold row represents the complete two-stage framework with the best overall performance. This configuration is used for the state-of-the-art comparison.

Configuration	False Positives per Scan							CPM
	0.125	0.25	0.5	1.0	2.0	4.0	8.0	
Improved YOLOX in Config D (WFF + GIoU + NWD)	0.842	0.889	0.902	0.906	0.910	0.910	0.910	0.896
Improved YOLOX in Config D + Kalman tracking ($N^{\prime }=3$)	0.878	0.897	0.912	0.927	0.941	0.949	0.950	0.922
**Improved YOLOX in Config D + Kalman tracking ($N^{\prime }=3$) + MIP + FP Classifier**	**0.865**	**0.911**	**0.926**	**0.941**	**0.955**	**0.967**	**0.977**	**0.934**

**Table 4:** Comparison with state-of-the-art methods.

Method	False Positive per Scan							CPM
	0.125	0.25	0.5	1.0	2.0	4.0	8.0	
Zheng et al. [34]	0.8760	0.8990	0.9120	0.9270	0.9420	0.9480	0.9530	0.9220
Cao et al. [47]	0.8480	0.8990	0.9250	0.9360	0.9490	0.9570	0.9600	0.9250
Ozdemir et al. [48]	0.8320	0.8790	0.9200	0.9420	0.9510	0.9590	0.9640	0.9210
Gong et al. [49]	0.7840	0.8470	0.9060	0.9380	0.9500	0.9550	0.9610	0.9060
Guo et al. [50]	0.8320	0.8860	0.9280	0.9370	0.9460	0.9520	0.9590	0.9200
Zhu et al. [51]	0.8500	0.8880	0.9270	0.9430	0.9520	0.9610	0.9710	0.9270
Zhang et al. [52]	0.8920	0.9140	0.9210	0.9250	0.9340	0.9410	0.9450	0.9240
Jiang et al. [53]	0.8101	0.8494	0.9003	0.9311	0.9406	0.9439	0.9473	0.9032
Ours	0.8651	0.9107	0.9255	0.9409	0.9548	0.9670	0.9769	0.9344

**Table 5:** Precision, Recall, and F1-score of the complete framework at different confidence thresholds.

Confidence Threshold	Recall	Precision	F1-Score	FP/Scan
0.0001	0.9615	0.3205	0.4759	3.9098
0.001	0.9615	0.3647	0.5280	3.2131
0.01	0.9573	0.5346	0.6832	1.5984
0.1	0.9316	0.7543	0.8330	0.5820
0.2	0.9231	0.8340	0.8765	0.3525
0.3	0.9017	0.8508	0.8755	0.3033
0.5	0.8547	0.9009	0.8772	0.1803

## Data Availability

The LIDC-IDRI dataset is publicly available at The Cancer Imaging Archive (TCIA): https://www.cancerimagingarchive.net/collection/lidc-idri/ (accessed on 13 August 2026). The LUNA16 annotation data are available at https://luna16.grand-challenge.org/ (accessed on 13 August 2026).

## Abbreviations

The following abbreviations are used in this manuscript:

CT	Computed tomography
LDCT	Low-dose computed tomography
CAD	Computer-aided diagnosis
HOG	Histogram of oriented gradients
LBP	Local binary patterns
GLCM	Gray-level co-occurrence matrix
CNN	Convolutional neural network
2D	Two-dimensional
3D	Three-dimensional
CBAM	Convolutional block attention module
ASPP	Atrous spatial pyramid pooling
CoT	Contextual transformer
GSA	Group shuffle attention
MIP	Maximum intensity projection
WFF-FPN	Weighted feature fusion-feature pyramid network
GIoU	Generalized intersection over union
NWD	Normalized wasserstein distance
YOLOX	You only look once X (network model)
CSP	Cross-stage partial
SPP	Spatial pyramid pooling
CBS	Convolution, batch normalization, silu
PANet	Path aggregation network
SimOTA	Simplified optimal transport assignment
BiFPN	Bidirectional feature pyramid network
CNT	Candidate nodule tracking
FROC	Free-response receiver operating characteristic
CPM	Competition performance metric
TP	True positives
FN	False negatives
FPs	False positives
SGD	Stochastic gradient descent
LUNA16	Lung nodule analysis 2016
LIDC-IDRI	The lung image database consortium and image database resource initiative
NIH	National institutes of health
